# Qili Qiangxin Capsule Combined With Sacubitril/Valsartan for HFrEF: A Systematic Review and Meta-Analysis

**DOI:** 10.3389/fphar.2022.832782

**Published:** 2022-04-04

**Authors:** Qian Xiang, Mengxi Wang, Yuhan Ding, Manlu Fan, Huaqin Tong, Jiandong Chen, Peng Yu, Le Shen, Xiaohu Chen

**Affiliations:** ^1^ Department of Cardiology, Affiliated Hospital of Nanjing University of Chinese Medicine, Nanjing, China; ^2^ Department of Cardiology, Jiangsu Province Hospital of Chinese Medicine, Nanjing, China; ^3^ First Clinical Medical College, Nanjing University of Chinese Medicine, Nanjing, China

**Keywords:** Qili Qiangxin capsule, heart failure with reduced ejection fraction, ARNI, sacubitril/valsartan, systematic review, meta-analysis

## Abstract

**Background:** Heart failure with reduced ejection fraction (HFrEF) is a complex, chronic disease and is among the top causes of morbidity and mortality. Angiotensin receptor-neprilysin inhibitor drugs represented by sacubitril/valsartan are the key drugs for the treatment of HFrEF in western medicine, and Qili Qiangxin Capsule (QQC) is a vital drug for the treatment of HFrEF in Chinese medicine. In recent years, there have been many relevant clinical studies on the combination of the two in the treatment of HFrEF. There are no systematic reviews or meta-analyses specific to sacubitril/valsartan combined with QQC for the treatment of HFrEF, so there is an urgent need to evaluate the effectiveness and safety of these two drugs.

**Objective:** To systematically assess the safety and effectiveness of QQC combined with sacubitril/valsartan in the treatment of HFrEF through a meta-analysis.

**Methods:** Searching studies on the combination of QQC and sacubitril/valsartan in the treatment of HFrEF, from databases such as PubMed, Cochrane Library, Web of Science, Wanfang Databases, Chinese Biomedical Literature Database, China Science and Technology Journal Database, and China National Knowledge Infrastructure, prior to 31 October 2021. Two reviewers regulated research selection, data extraction, and risk of bias assessment. Review Manager Software 5.4 was used for meta-analysis.

**Results:** There were 26 studies with 2,427 patients included in total. The meta-analysis showed the combination therapy has significant advantages in improving the clinical efficacy, 6-MWT (RR = 1.18, 95% CI: 1.11–1.26, MD = 70.65, 95% CI: 23.92–117.39), superior in ameliorating LVEF, LVEDD, LVESD, and SV (LVEF: MD = 5.41, 95% CI: 4.74–6.08; LVEDD: MD = −4.41, 95% CI: −6.19 to −2.64; LVESD: MD = −3.56, 95% CI: −4.58 to −2.54; and SV: MD = 5.04, 95% CI: 3.67–6.40), and in improving BNP, NT-proBNP, AngII, and ALD (BNP: MD = −97.55, 95% CI: −112.79 to −82.31; NT-proBNP: MD = −277.22, 95% CI: −348.44 to −206.01; AngII: MD = −11.48, 95% CI: −15.21 to −7.76; and ALD: MD = −26.03, 95% CI: −38.91 to −13.15), and all the differences have statistical advantages (p < 0.05). There are no advantages in improving CO and adverse events (MD = 0.66, 95% CI: −0.12 to 1.43 and RR = 0.62, 95% CI: 0.37–1.04, respectively), and the differences have no statistical advantages.

**Conclusion:** Compared with the control group, QQC combined with sacubitril/valsartan may be effective in the treatment of HFrEF. However, the conclusion of this study must be interpreted carefully due to the high risk and ambiguity of bias in the included trials.

## Introduction

Heart failure (HF) is the symptoms and signs caused by abnormal heart structure or function, usually accompanied by elevated natriuretic peptide levels and/or objective evidence of cardiogenic pulmonary circulation or systemic congestion. The objective evidence includes imaging clinical syndromes such as physical examination, resting, or load hemodynamic monitoring. In 2016, European Society of Cardiology (ESC) divided HF into three types: HF with preserved ejection fraction (HFpEF, LVEF > 50%), HF with midrange ejection fraction (HFmrEF, LVEF 40%–49%), and HF with reduced ejection fraction (HFrEF, LVEF < 40%) (Crea, 2021). A report based on the global burden of disease showed that the number of patients with HF has increased from 33.5 million in 1990 to 64.3 million in 2017 (Bragazzi et al., 2021). The increase almost doubled and continued to grow rapidly due to aging populations. According to the “China Cardiovascular Disease Report 2018,” there are about 5 million HF patients in China. The 5-year average survival rate is about 50%, and the 10-year average survival rate is only 10% (Hu et al., 2019).

Past evidence has shown that the Frank–Starling mechanism, myocardial hypertrophy, and neurohumoral compensation mechanism are the key compensatory mechanisms leading to HF (Toischer et al., 2010). Drugs such as renin–angiotensin system inhibitors, sympathetic adrenergic system inhibitors, and aldosterone receptor antagonists were used to treat HF (Teerlink et al., 2017). The CCS/CHFS Heart Failure Guidelines Update released in 2021 recommends, on the basis of existing evidence, angiotensin receptor-neprilysin inhibitor (ARNI) to replace ACEI/ARB and added sodium-dependent glucose transporters 2 (SGLT2) drugs represented by sacubitril/valsartan (Bozkurt et al., 2021).

Qili Qiangxin Capsule (QQC) is a drug recommended in the “Chinese Heart Failure Diagnosis and Treatment Guidelines 2018” (Wang and Liang, 2018). The main components of QQC are *Astragalus*, Tinglizi, Danshen, Aconite, Ginseng, *Polygonatum odoratum*, *Alisma orientalis*, dried tangerine peel, Xiangjiapi, safflower, and cinnamon sticks. QQC can reduce the high expression of MMP-2 and MMP-9 in cardiomyocytes, the activation of the RAAS system in the paraventricular nucleus of the hypothalamus, and also the activity of renal sympathetic nerves, improve the heart function of rats with chronic heart failure, and delay the progression of HF (Ma et al., 2016).

In recent years, a large number of clinical trials have shown that QQC has a definite effect on the treatment of HFrEF. Because sacubitril/valsartan is a treatment drug clearly recommended by the latest guidelines, there is currently a lack of meta-analysis of QQC combined with sacubitril/valsartan in the treatment of HFrEF. This study aims to systematically evaluate the safety and effectiveness of QQC combined with sacubitril/valsartan in the treatment of HFrEF.

## Methods

### Search Strategy

PubMed, Cochrane Library, Web of Science, Wanfang Databases, Chinese Biomedical Literature Database (CBM), China Science and Technology Journal Database (VIP), and China National Knowledge Infrastructure (CNKI) were searched, and RCT studies of QQC combined with sacubitril/valsartan in the treatment of HFrEF were collected from the date of establishment of each database to 31 October 2021. No language restrictions were imposed. The search terms included “Qili Qiangxin capsule,” “Qili Qiangxin,” “sacubitril/valsartan,” “sacubitril and valsartan,” “ARNI,” “chronic heart failure,” “heart failure with reduced ejection fraction,” “cardiac failure,” “heart decompensation,” and “HFrEF.”

### Study Inclusion and Exclusion Criteria

Only RCTs will be included, and the subjects of the included study must meet the diagnostic criteria for chronic heart failure. The intervention should be QQC combined with sacubitril/valsartan. The control group should be sacubitril/valsartan, regardless of whether both groups combined with other western medicine treatments. Moreover, the outcomes included clinical efficiency rates, left ventricular ejection fractions (LVEF), left ventricular end-diastolic dimension (LVEDD), left ventricular end-Systolic diameter (LVESD), N-terminal pro-B-type natriuretic peptide (NT-proBNP), brain natriuretic peptide (BNP), 6-minute walking distance (6-MWD), cardiac output (CO), stroke volume (SV), aldosterone (ALD), angiotensin II (Ang-Ⅱ), and the adverse events. The exclusion criteria included studies with incomplete data, repeated publications, or mismatched outcome indicators.

### Data Extraction and Quality Assessment

Two researchers separately extracted literature data, including the author, publication year, sample size, age, gender, intervention measures, treatment methods, and treatment time. In the event of a disagreement, a third party participated in the negotiation and settlement. A systematic review was conducted according to the Cochrane Collaboration handbook. The evaluation includes seven types: random sequence generation, incomplete outcome data, allocation concealment, selective reporting, blinding of participants and personnel, blinding of outcome assessment, and for-profit bias. Then, Review Manager 5.4 should be used to display the bias risk assessment chart drawn.

### Data Analysis

RevMan 5.4 software was used for meta-analysis. Binary variables were statistically analyzed by risk ratio (RR), and continuous variables were statistically analyzed by mean difference (MD). Each effect size was evaluated with a 95% confidence interval (CI). The statistical heterogeneity was measured according to the value of *I* ^*2*^, and the evaluation results are shown in forest maps. The random effects model was used for analysis if *I* ^*2*^ ≥ 50%. Otherwise, the fixed effects model was used for analysis. Considering that the dose of the drug may be the source of heterogeneity, the statistical analysis is based on the dose of sacubitril/valsartan for subgroup analysis, and the subgroup was divided into three groups: the dosage of sacubitril/valsartan 100 mg twice a day, 200 mg twice a day, and other doses. Finally, a bias test is performed on the effective rate, and the results are displayed in a funnel chart.

## Results

### Search Results and Included Studies

**FIGURE 1:**
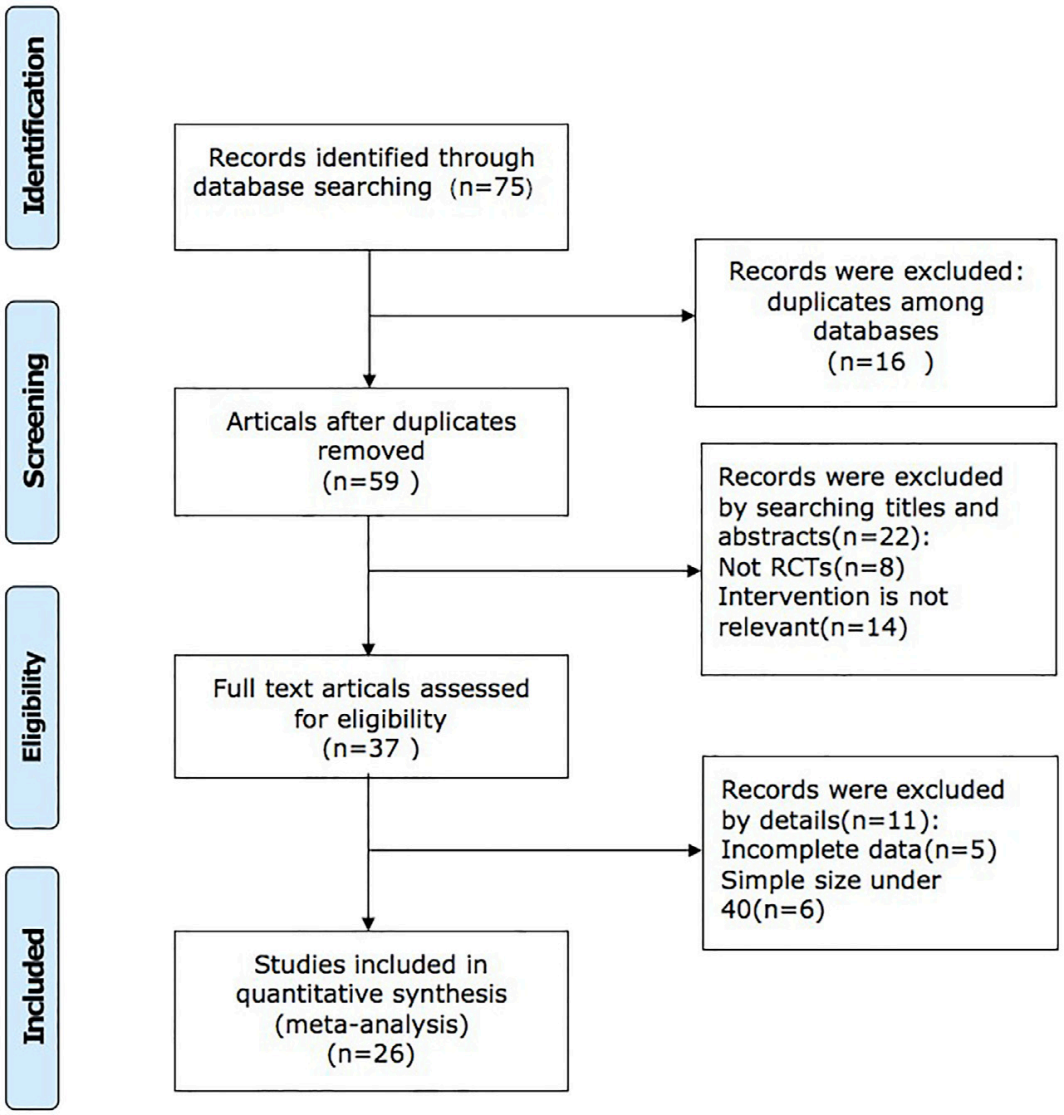
Flow diagram of study selection and identification.

We retrieved 75 studies, and 26 full-text articles pertaining to 2,427 patients which were eventually included based on inclusion and exclusion criteria (Brewster et al., 2003; McMurray et al., 2013; Shi, 2018; Wang, 2019; Dong and Du, 2020; Gao et al., 2020; Huang et al., 2020; Li et al., 2020; Liu, 2020; Qin et al., 2020; Qu, 2020; Su et al., 2020; Wang et al., 2020; Xu, 2020; Zhang, 2020; Zhao et al., 2020a; Zhao et al., 2020b; Han, 2021; Lin et al., 2021; Ma et al., 2021; Su and Sun, 2021; Tan, 2021; Wang, 2021; Wang et al., 2021; Wang and Shang, 2021; Yang, 2021; Yao and Li, 2021; Zhang, 2021). The study selection process is shown in Figure 1. All of the trials were conducted in China and published in Chinese. Except for the control group in one of the studies, which was valsartan combined with western medicine, the control group in the other studies was sacubitril/valsartan combined with western medicine or not. The dosage of QQC in the interventional group was 0.3g, three times a day, and the dosages of sacubitril/valsartan and conventional Western medicine treatment were not limited in both interventional and control groups. Characteristics of included trials are shown in Table 1.

**TABLE 1:** Characteristics of included studies.

Study ID	Sample size (T/C)	Intervention (T/C)	Age (T/C)	Gender (M/F)	Treatment duration	Drug dosage	Outcomes
Dong and Du (2020)	60/60	I + II + III/II + III	68.9 ± 4.56/71.3 ± 5.62	78/42	NR	Ⅰ: 0.3 g tid; Ⅱ: 200 mg bid	②③⑤
Gao et al. (2020)	51/51	I + II/II	64.23 ± 6.07/7.02 ± 3.98	64/38	6 m	Ⅰ: 0.3 g tid; Ⅱ: 200 mg bid	①②③④⑤⑪⑬
Han (2021)	34/34	I + II/II	66.44 ± 5.66/67.88 ± 6.87	39/29	6 m	Ⅰ: 0.3 g tid; Ⅱ: 200 mg bid	①②③④⑩
Huang et al. (2020)	155/150	I + II + III/III + valsartan	68.52 ± 4.67/68.58 ± 4.73	161/144	10 w	Ⅰ: 0.3 g tid; Ⅱ: 50 mg bid	①②⑥⑦⑩
Li et al. (2020)	35/35	I + II + III/II + III	38.5 ± 4.1/35.2 ± 3.9	31/39	8 w	Ⅰ: 0.3 g tid; Ⅱ: 50 mg qd	①②③⑥⑩⑪
Lin et al. (2021)	41/42	I + II + III/II + III	62.42 ± 7.96/62.42 ± 7.96	41/42	12 w	Ⅰ: 0.3 g tid; Ⅱ: 50 mg bid	①②⑧⑨
Liu (2020)	38/42	I + II + III/II + III	71.52 ± 6.46/71.58 ± 6.39	61/19	1 m	Ⅰ: 0.3 g tid; Ⅱ: 100 mg bid	②③④
Ma et al. (2021)	56/56	I + II + III/II + III	65.45 ± 10.51/66.17 ± 9.79	62/50	12 w	Ⅰ: 0.3 g tid; Ⅱ: 100 mg bid	①②③⑦⑩⑪⑬
Qin et al. (2020)	47/47	I + II/II	70.00 ± 2.32/69.88 ± 2.48	66/28	3 m	Ⅰ: 0.3 g tid; Ⅱ: 50 mg bid	②③④⑤⑪⑫
Qu (2020)	34/34	I + II + III/II + III	65.34 ± 3.53/65.26 ± 3.46	38/30	30 d	Ⅰ: 0.3 g tid; Ⅱ: 200 mg bid	①②③⑩
Shi (2018)	38/38	I + II + III/II + III	71.5 ± 4.8/72.3 ± 5.3	54/22	4 w	Ⅰ: 0.3 g tid; Ⅱ: 50 mg bid	①②④
Su et al. (2020)	37/37	I + II + III/II + III	59.06 ± 8.52/58.39 ± 8.06	43/31	NR	Ⅰ: 0.3 g tid; Ⅱ: 200 mg bid	①②⑤⑦⑬
Su and Sun (2021)	34/34	I + II + III/II + III	65.2 ± 7.3/62.3 ± 7.9	NR	NR	Ⅰ: 0.3 g tid; Ⅱ: 100 mg qd	①⑥
Tan (2021)	40/40	I + II + III/II + III	59.15 ± 8.43/59.21 ± 8.12	47/33	3 m	Ⅰ: 0.3 g tid; Ⅱ: 100 mg bid	①③⑤
Wang and Shang (2021)	28/27	I + II + III/II + III	54.1 ± 11.4/54.6 ± 11.0	32/23	8 w	Ⅰ: 0.3 g tid; Ⅱ: 100 mg bid	①②③⑪
Wang et al. (2021)	40/40	I + II + III/II + III	NR	55/25	NR	Ⅰ: 0.3 g tid; Ⅱ: 50 mg bid	①②④⑧⑨
Wang (2019)	57/57	I + II + III/II + III	63.74 ± 5.7/63.74 ± 5.7	63/51	1 m	Ⅰ: 0.3 g tid; Ⅱ: 50 mg bid	①②④
Wang et al. (2020)	45/41	I + II + III/II + III	54.4 ± 10.8/53.9 ± 11.3	57/29	8 w	Ⅰ: 0.3 g tid; Ⅱ: 100 mg bid	①②③④⑪⑫
Xu (2020)	50/50	I + II + III/II + III	53.79 ± 4.46/54.13 ± 7.42	49/51	12 w	Ⅰ: 0.3 g tid; Ⅱ: 100 mg bid	①②③④⑤⑧⑨⑪⑫
Yang (2021)	43/43	I + II + III/II + III	62.64 ± 1.23/62.58 ± 1.17	47/39	3 m	Ⅰ: 0.3 g tid; Ⅱ: 100 mg qd	①⑤⑩
Wang (2021)	50/50	I + II + III/II + III	46.82 ± 0.19/47.47 ± 0.32	47/53	NR	Ⅰ: 0.3 g tid; Ⅱ: 25 mg qd	②③⑥
Yao and Li (2021)	20/20	I + II+ III/II + III	62.13 ± 3.16/61.28 ± 3.27	23/17	1 m	Ⅰ: 0.3 g tid; Ⅱ: 200 mg bid	①②④⑦⑬
Zhang, (2021)	41/41	I + II + III/II + III	64.86 ± 6.43/65.42 ± 6.19	47/35	4 w	Ⅰ: 0.3 g tid; Ⅱ: 50 mg bid	①②③④⑫
Zhang (2020)	44/44	I + II/II	76.5 ± 9.8/75.5 ± 9.4	49/39	30 d	Ⅰ: 0.3 g tid; Ⅱ: 50 mg bid	①②④
Zhao et al. (2020a)	51/51	I + II + III/II + III	62.4 ± 2.5/65.3 ± 2.7	56/46	12 w	Ⅰ: 0.3 g tid; Ⅱ: 100 mg bid	①②③⑤⑪
Zhao et al. (2020b)	47/47	I + II/II	58.6 ± 6.5/58.7 ± 6.5	54/40	8 w	Ⅰ: 0.3 g tid; Ⅱ: 200 mg bid	①②③⑪

T, test; C, control; NR, not reported; I, QQC; Ⅱ, sacubatril valsartan; Ⅲ, conventional treatment; d, day (s); w, week(s); m, month(s); ① clinical efficiency; ② LVEF; ③ LVEDD; ④ 6-MWT; ⑤ NT-proBNP; ⑥ BNP; ⑦ SV; ⑧ AngⅡ; ⑨ ALD; ⑩ adverse events; ⑪ LVESD; ⑫ MLHFQ; ⑬ CO.

### Risk of Bias in Included Trials

**FIGURE 2:**
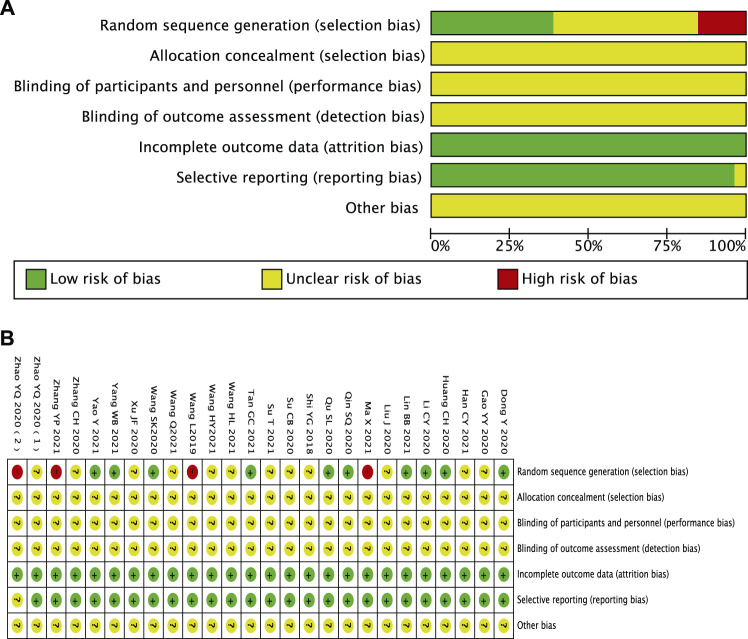
Assessment of risk of bias. **(A)** Risk of bias graph and **(B)** risk of bias summary.

Ten studies reported methods of randomizing participants using random number tables, which were considered to be low risk of bias (Dong and Du, 2020; Huang et al., 2020; Li et al., 2020; Qin et al., 2020; Qu, 2020; Wang et al., 2020; Han, 2021; Lin et al., 2021; Wang and Shang, 2021; Yang, 2021; Yao and Li, 2021), and 12 trials were considered to be an unclear risk of bias because they did not introduce the randomization method in detail (Shi, 2018; Gao et al., 2020; Liu, 2020; Su et al., 2020; Xu, 2020; Zhang, 2020; Zhao et al., 2020b; Han, 2021; Su and Sun, 2021; Wang, 2021; Wang et al., 2021; Wang and Shang, 2021). One study did not mention randomization (Wang, 2019), and three studies were grouped according to the date of visit and intervention measures (Zhao et al., 2020a; Ma et al., 2021; Zhang, 2021). These four trials were identified as high-risk bias. All studies did not report adequate allocation concealment and were blinded to participants or outcome evaluators, which were considered as unclear risk of bias. All studies have no patients falling off and reported test indicators as planned. There was no selective reporting of research results. It was unclear whether there is other bias (Figure 2).

### Clinical Efficiency Rates

**FIGURE 3:**
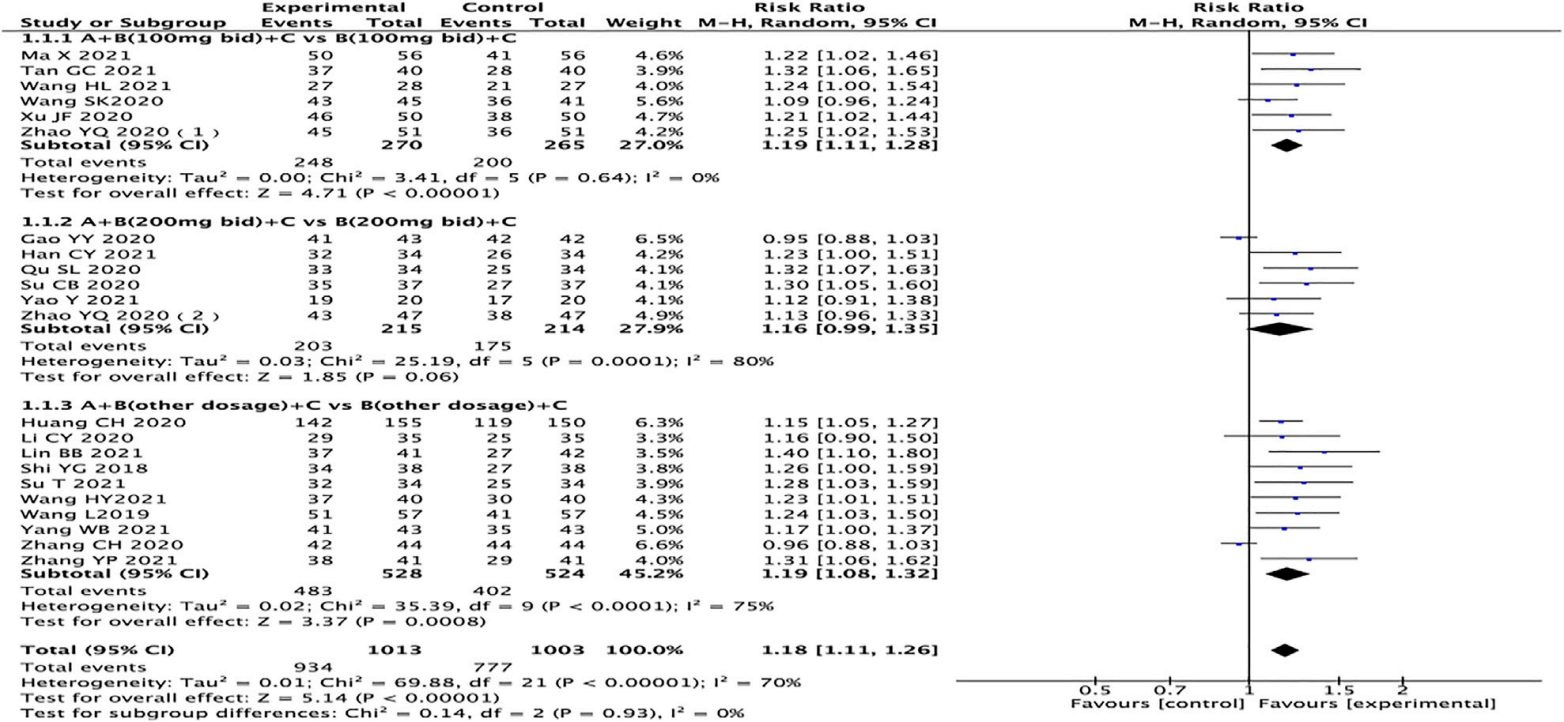
Forest plot of the clinical efficacy rates.

A total of 22 studies reported clinical efficacy, with a total of 2016 patients (Toischer et al., 2010; Ma et al., 2016; Teerlink et al., 2017; Shi, 2018; Wang and Liang, 2018; Hu et al., 2019; Wang, 2019; Zhao et al., 2020a; Zhao et al., 2020b; Gao et al., 2020; Huang et al., 2020; Li et al., 2020; Qu, 2020; Su et al., 2020; Wang et al., 2020; Xu, 2020; Zhang, 2020; Bozkurt et al., 2021; Bragazzi et al., 2021; Crea, 2021; Han, 2021; Lin et al., 2021; Ma et al., 2021; Su and Sun, 2021; Tan, 2021; Wang et al., 2021; Wang and Shang, 2021; Yang, 2021; Yao and Li, 2021; Zhang, 2021). The heterogeneity test results suggest that the random effects model should be used for meta-analysis (*I* ^*2*^ = 70%, *p* < 0.00001), and the subgroup analysis is performed according to the different dosages of sacubitril/valsartan. The outcome shows that the experimental group is significantly superior in terms of total clinical effective rates (RR = 1.18, 95% CI: 1.11–1.26, *p <* 0.00001). Regardless of the dosage of sacubitril/valsartan, the test group is also better than the control group (RR: 1.19, 95% CI: 1.11–1.28; RR: 1.16, 95% CI: 0.99–1.35, RR: 1.19, 95% CI:1.08–1.32, for the dosages of sacubitril/valsartan of 100 mg, 200 mg, and other doses, respectively) (Figure 3).

### 6-MWT

**FIGURE 4:**
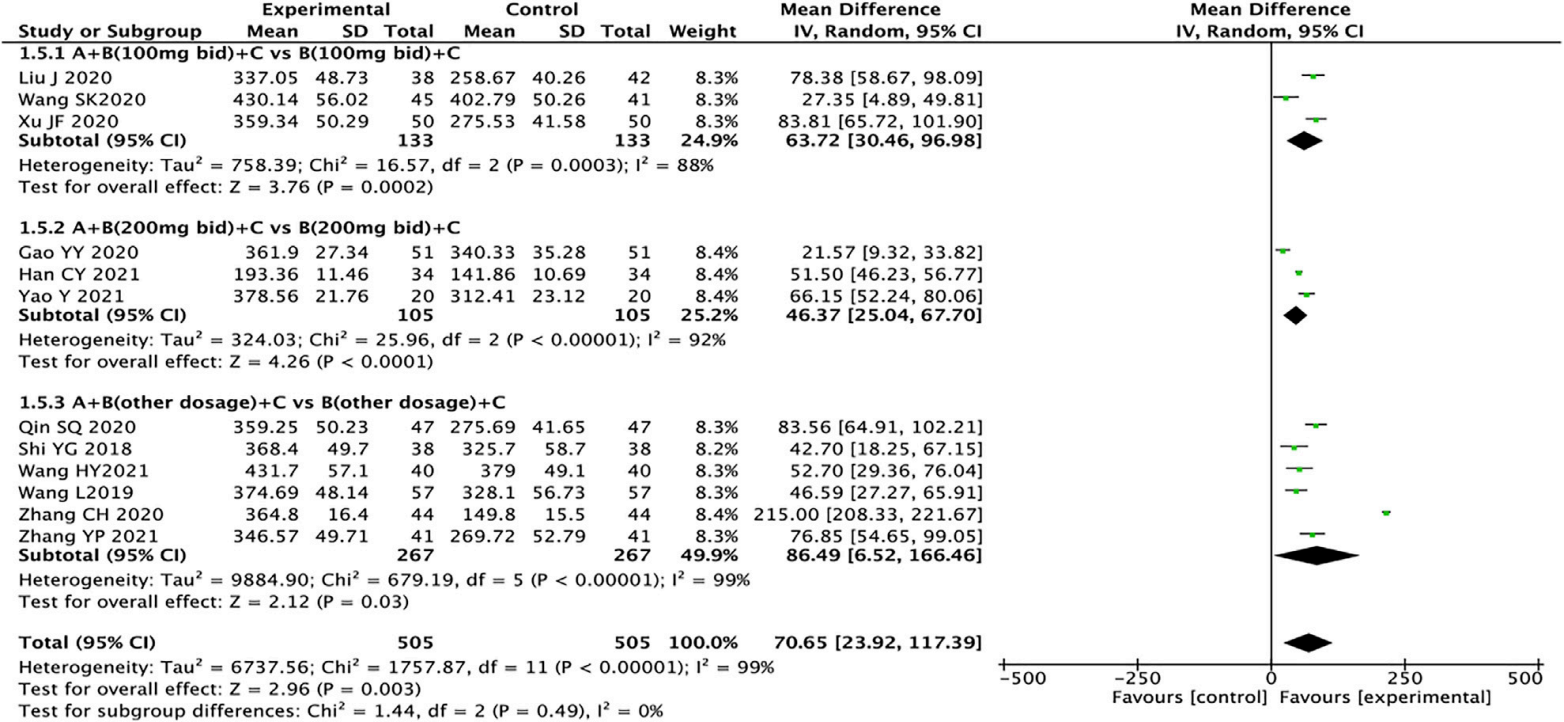
Forest plot of 6-MWT.

A total of 12 articles reported 6-MWT, with a total of 1,010 patients (Shi, 2018; Wang, 2019; Gao et al., 2020; Huang et al., 2020; Liu, 2020; Qin et al., 2020; Wang et al., 2020; Xu, 2020; Zhang, 2020; Han, 2021; Wang et al., 2021; Yao and Li, 2021; Zhang, 2021). The heterogeneity test results suggest that the random effects model should be used for meta-analysis (*I* ^*2*^ = 99%, *p* < 0.00001). Subgroup analysis is still performed based on the dosage of sacubitril/valsartan. The results showed that the experimental group had significant advantages in ameliorating the 6-MWT more than the control group (MD = 70.65, 95% CI: 23.92–117.39, *p* = 0.003). Regardless of the dosage of sacubitril/valsartan, the test group is also better than the control group (MD: 63.72, 95% CI: 30.46–96.98; MD: 46.37, 95% CI: 25.04–67.70, MD: 86.49, 95% CI: 6.52–166.46, for the dosages of sacubitril/valsartan of 100 mg, 200 mg, and other doses, respectively). The results of subgroup analysis indicated that the dose of sacubitril/valsartan may not be the source of heterogeneity, and we speculated that the source of heterogeneity might be related to factors such as the severity of the patient’s disease and the duration of treatment (Figure 4).

### Left Ventricular Ejection Fractions

**FIGURE 5:**
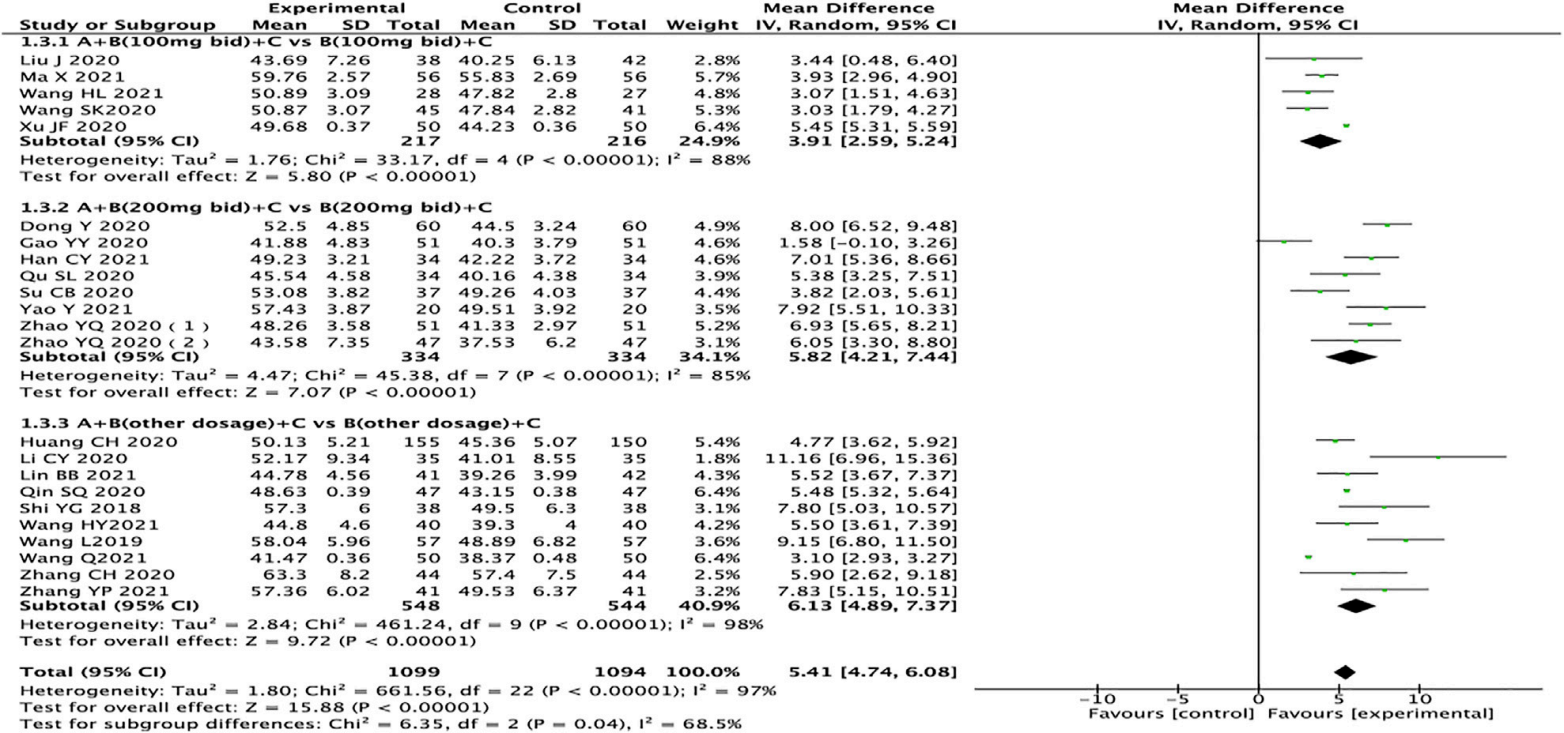
Forest plot of LVEF.

A total of 23 articles reported LVEF, with a total of 2,193 patients (Shi, 2018; Wang, 2019 Dong and Du, 2020; Gao et al., 2020; Huang et al., 2020; Li et al., 2020; Liu, 2020; Qin et al., 2020; Qu, 2020; Su et al., 2020; Wang et al., 2020; Xu, 2020; Zhang, 2020; Zhao et al., 2020a; Zhao et al., 2020b; Han, 2021; Lin et al., 2021; Ma et al., 2021; Wang, 2021; Wang et al., 2021; Wang and Shang, 2021; Yao and Li, 2021; Zhang, 2021). The heterogeneity test results suggest that the random effects model should be used for meta-analysis (*I* ^*2*^ = 97%, *p <* 0.00001). Subgroup analysis is still performed based on the dosage of sacubitril/valsartan. The results showed that compared with the control group, the experimental group had significant advantages in improving the LVEF of HFrEF patients (MD = 5.41, 95% CI:4.74 to 6.08, *p <* 0.00001). Regardless of the dosage of sacubitril/valsartan, the test group is also better than the control group (MD: 3.91, 95% CI: 2.59–5.24; MD: 5.82, 95% CI: 4.21–7.44; MD: 6.13, 95% CI: 4.89–7.37, for the dosages of sacubitril/valsartan of 100 mg, 200 mg, and other doses, respectively). The results of subgroup analysis indicated that the dose of sacubitril/valsartan may not be the source of heterogeneity, and we speculated that the source of heterogeneity might be related to the patient’s course of disease, course of treatment, and other factors (Figure 5).

### Left Ventricular End-Diastolic Dimension

**FIGURE 6:**
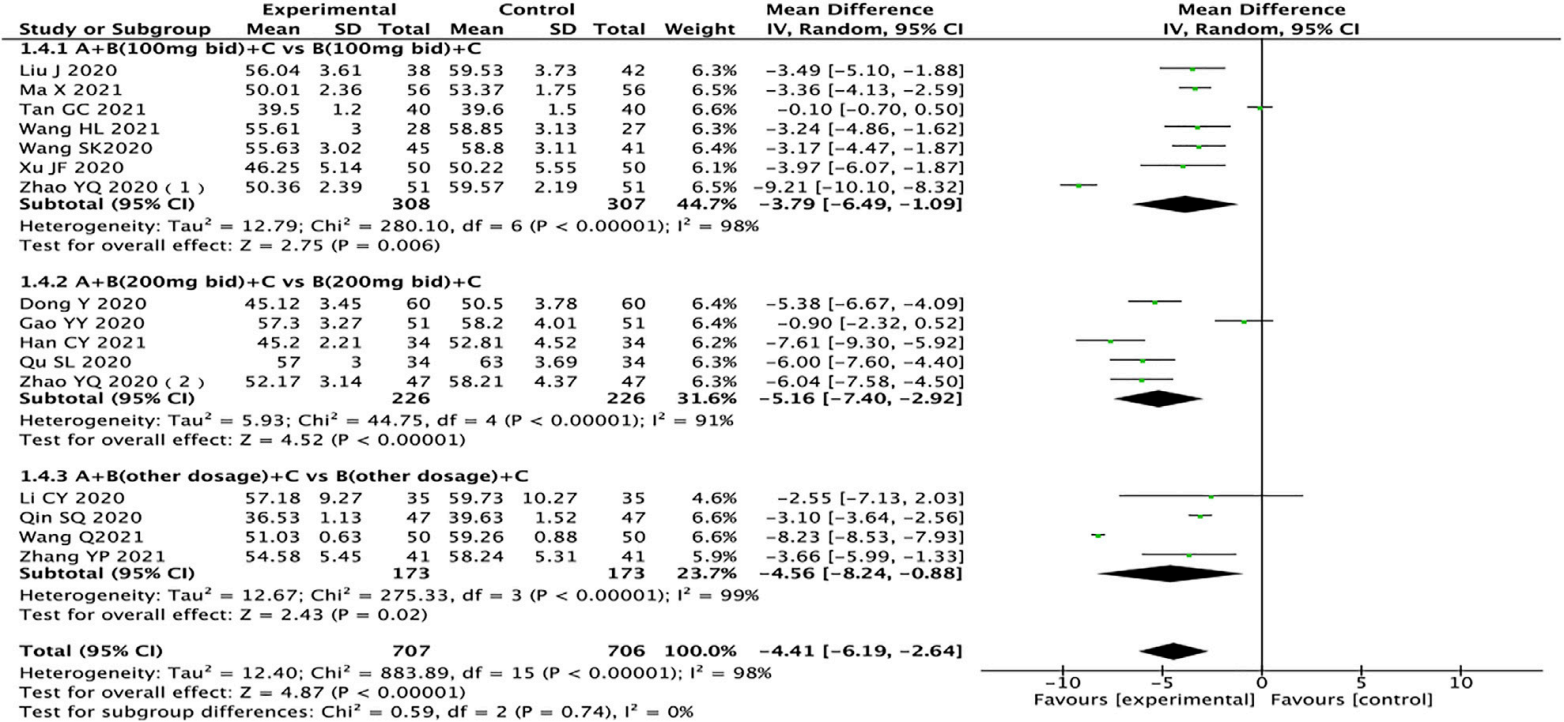
Forest plot of LVEDD.

A total of 16 studies pertaining to 1,413 patients reported LVEDD (Dong and Du, 2020; Gao et al., 2020; Li et al., 2020; Liu, 2020; Qin et al., 2020; Qu, 2020; Wang et al., 2020; Xu, 2020; Zhao et al., 2020a; Zhao et al., 2020b; Han, 2021; Ma et al., 2021; Su and Sun, 2021; Wang, 2021; Wang and Shang, 2021; Zhang, 2021). The results of subgroup analysis showed that drug doses might not be the source of heterogeneity. After excluding one study in each group (Gao et al., 2020; Tan, 2021; Wang, 2021), the heterogeneity between groups was reduced (*I* ^*2*^ < 50%). After analyzing the literature, it was considered that the source of heterogeneity might be related to the length of treatment. The heterogeneity test results showed that the random effects model should be used (*I* ^*2*^ = 98%, *p <* 0.00001). The results showed that compared with the control group, the test group has more advantages in the treatment of LVEDD in HFrEF patients (MD = −4.41, 95% CI: −6.19 to −2.64, *p <* 0.00001). Regardless of the dosage of sacubitril/valsartan, the test group is also better than the control group (MD: −3.79, 95% CI: −6.49 to −1.09; MD: −5.16, 95% CI: −7.40 to −2.92; MD: −4.56, 95% CI: −8.24 to −0.88, for 100 mg, 200 mg, other doses, respectively) (Figure 6).

### Left Ventricular End-Systolic Diameter

**FIGURE 7:**
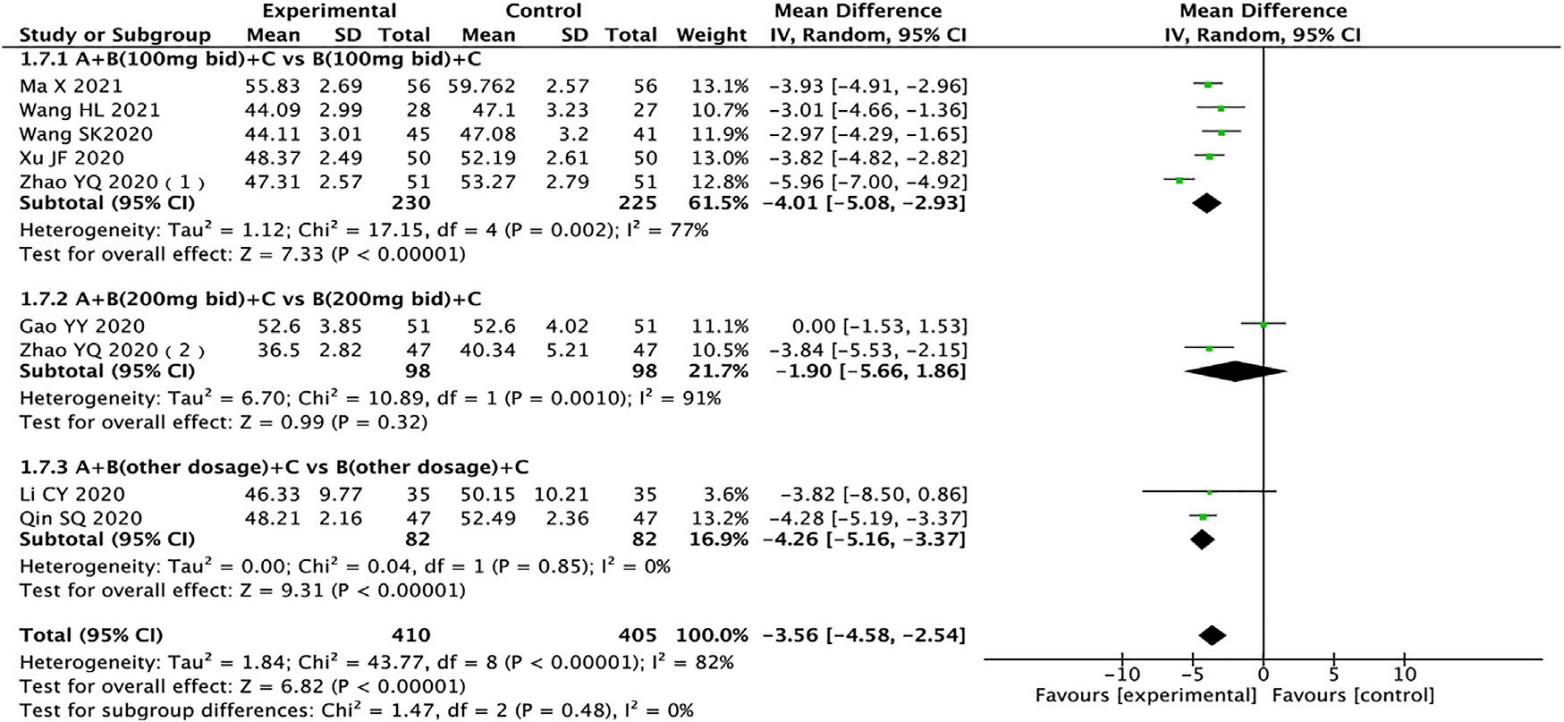
Forest plot of LVESD.

A total of nine trials containing 815 patients reported LVESD (Gao et al., 2020; Li et al., 2020; Qin et al., 2020; Wang et al., 2020; Xu, 2020; Zhao et al., 2020a; Zhao et al., 2020b; Ma et al., 2021; Wang and Shang, 2021). The heterogeneity test results showed that the random effects model should be used (*I* ^*2*^ = 82%, *p* < 0.00001), and subgroup analysis results showed that drug dosage might be the source of heterogeneity. The results showed that compared with the control group, the test group has more advantages in the treatment of LVESD in HFrEF patients (MD = −3.56, 95% CI: −4.58 to −2.54, *p <* 0.00001). When the dose of sacubitril/valsartan is 200 mg twice a day, the result of the test group is no better than the control group, which is not statistically significant (MD = −1.90, 95% CI: −5.66 to 1.86, *p* = 0.32) (Figure 7).

### N-Terminal Pro-B-Type Natriuretic Peptide

**FIGURE 8:**
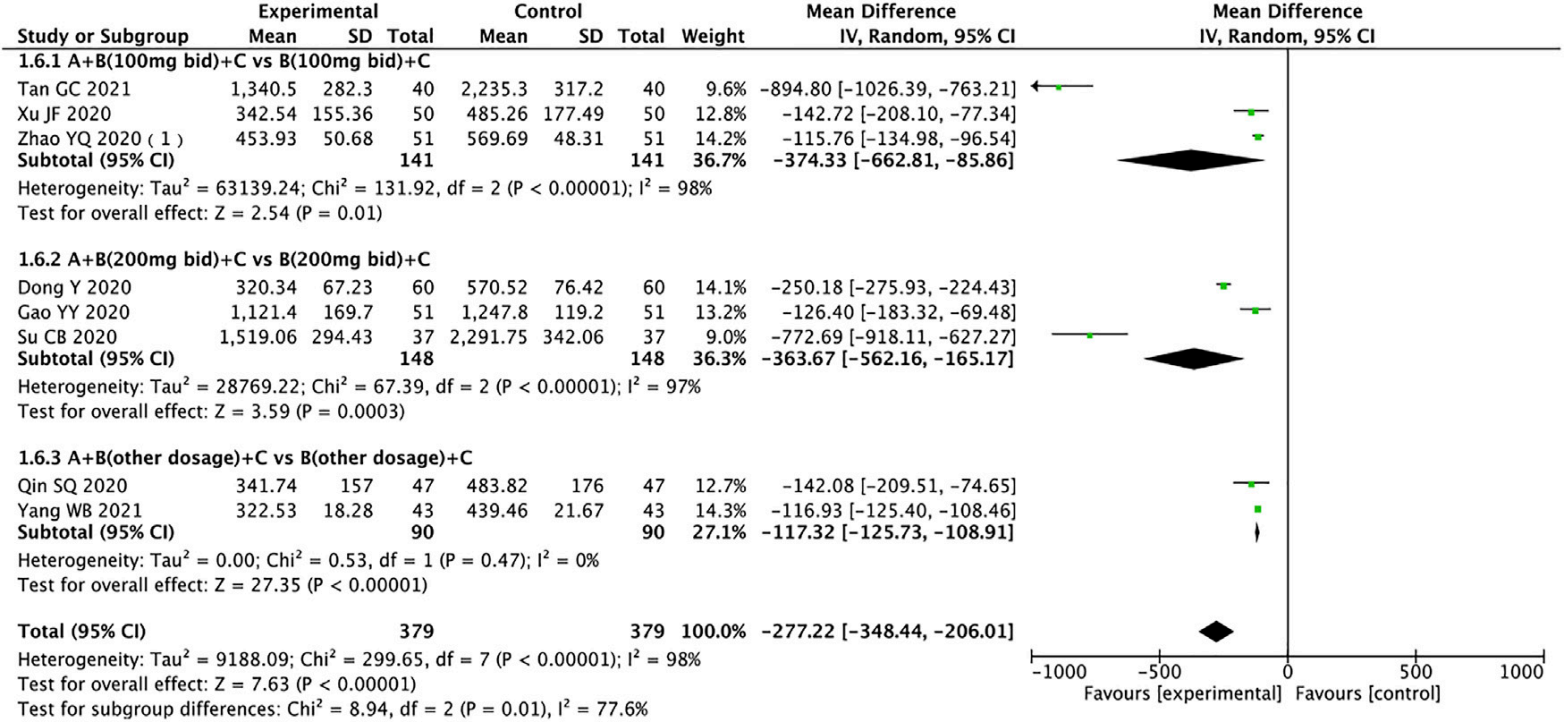
Forest plot of NT-proBNP.

A total of 758 patients from eight trials reported NT-proBNP (Dong and Du, 2020; Gao et al., 2020; Qin et al., 2020; Su et al., 2020; Xu, 2020; Zhao et al., 2020a; Tan, 2021; Yang, 2021). The random effects model was used for statistical analysis (*I* ^*2*^ = 98%, *p* < 0.00001), and subgroup analysis suggested that sources of heterogeneity may be drug dose, duration of treatment, or other factors. The results showed that the experimental group was significantly better than the control group in ameliorating NT-proBNP in HFrEF patients (MD = −277.22, 95% CI: −348.44 to −206.01, *p* < 0.00001). The experimental group also outperformed the control group regardless of the dose of sacubitril/valsartan (MD: −374.33, 95% CI: −662.81 to −85.86; MD: −363.67, 95% CI: −562.16 to −165.17; MD: −117.32, 95% CI: −125.73 to −108.91, for 100 mg, 200 mg, other doses, respectively) (Figure 8).

### Brain Natriuretic Peptide, AngⅡ, and Aldosterone

**FIGURE 9:**
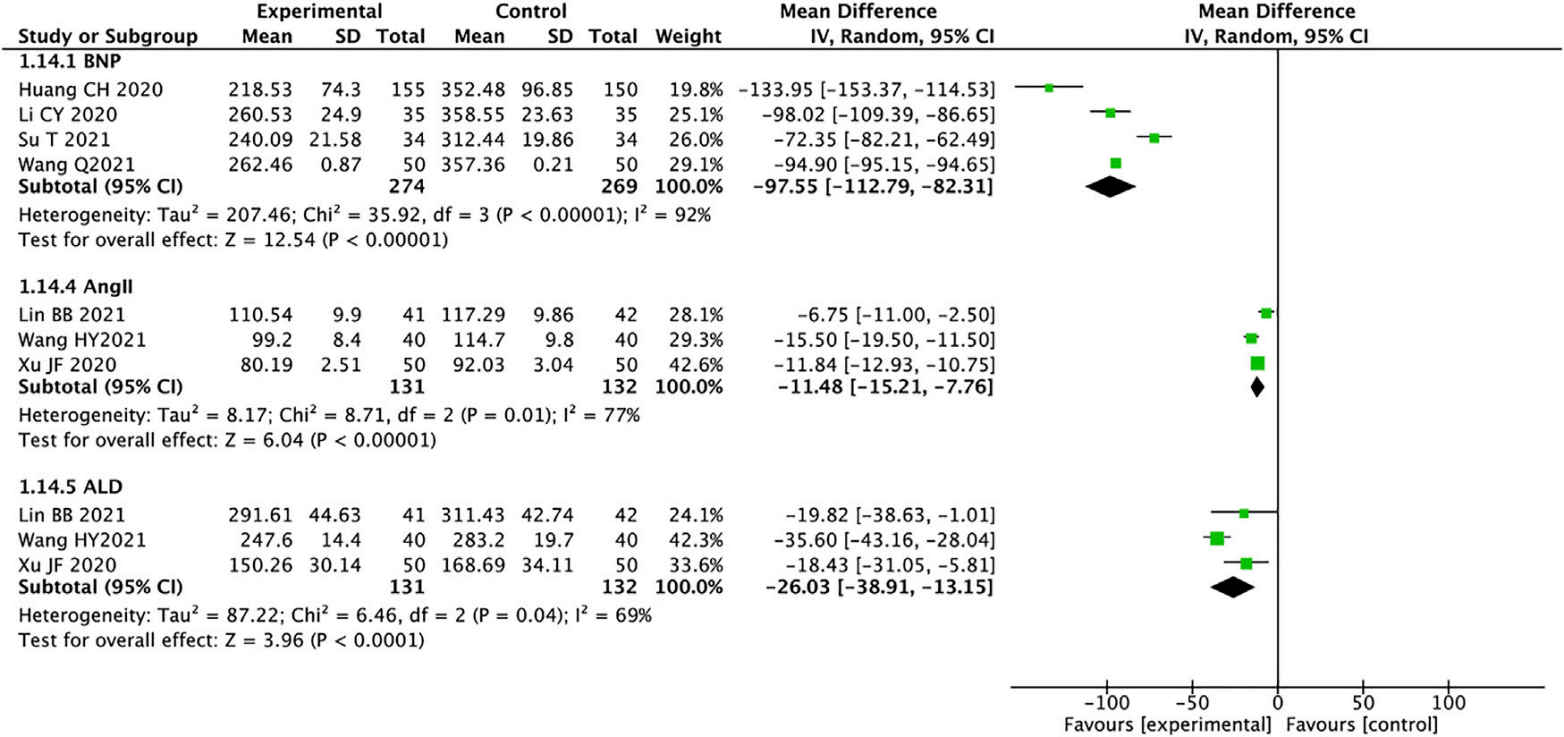
Forest plot of BNP, AngII, and ALD.

A total of four studies containing 543 patients reported on BNP (Huang et al., 2020; Li et al., 2020; Su and Sun, 2021; Wang, 2021). We used the random effects model for statistical analysis based on the heterogeneity (*I* ^*2*^ = 92%, *p* < 0.00001). The results showed that the experimental group was significantly better than the control group in improving the BNP of HFrEF patients (MD = −97.55, 95% CI: −112.79 to −82.31, *p* < 0.00001). Three trials containing 263 patients reported AngII (Liu, 2020; Xu, 2020; Lin et al., 2021), and the random effects model was used for analysis (*I* ^*2*^ = 77%, *p* = 0.01). MD = −11.48, 95% CI: −15.21 to −7.76, *p <* 0.00001, means that the experimental group had an advantage in improving the AngII of patients with HFrEF. A total of 263 patients from three trials reported ALD (Liu, 2020; Xu, 2020; Lin et al., 2021). The random effects model was used for statistical analysis (*I* ^*2*^ = 69%, *p* = 0.04). The results showed that the experimental group was significantly better than the control group in ameliorating ALD in HFrEF patients (MD = −26.03, 95% CI: −38.91 to −13.15, *p* < 0.0001) (Figure 9). We analyzed the source of heterogeneity for these outcomes, which may be related to patient age and disease severity.

### Stroke Volume and Cardiac Output

**FIGURE 10:**
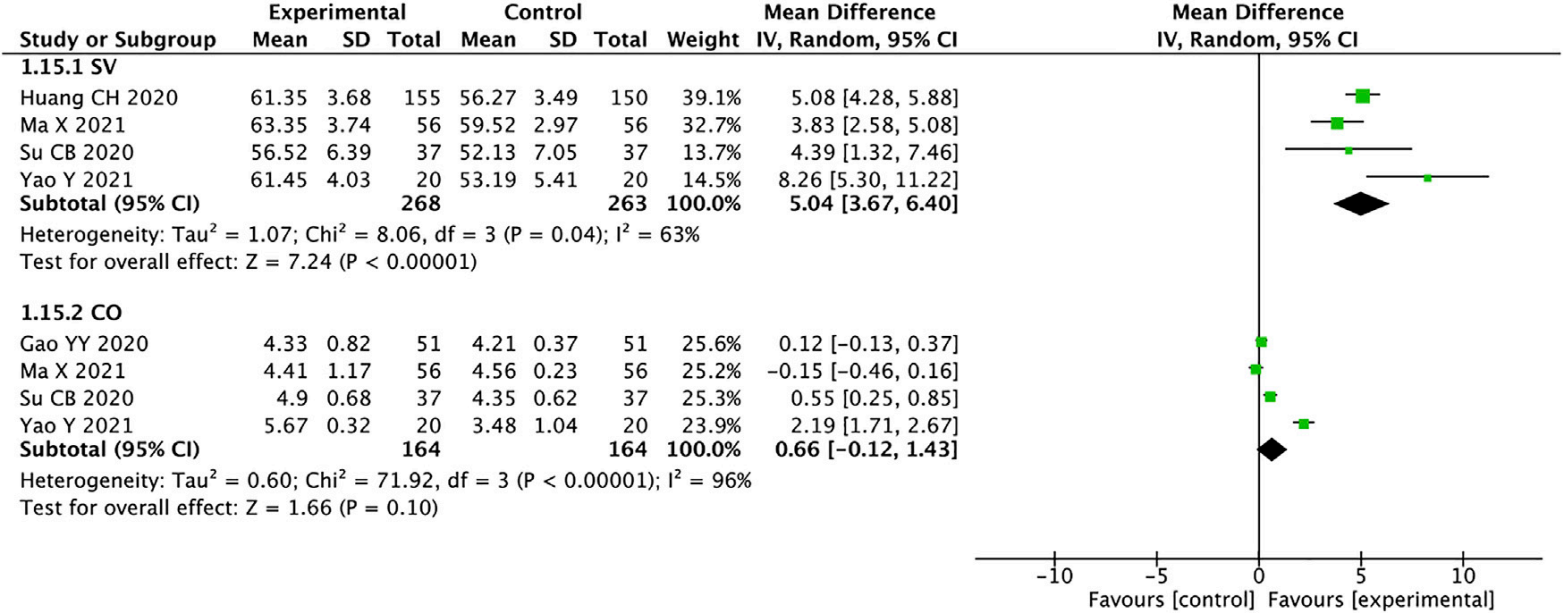
Forest plot of SV and CO.

A total of 531 patients from four studies were included in the analysis of SV(Huang et al., 2020; Su et al., 2020; Ma et al., 2021; Yao and Li, 2021), and we used random effects model for statistical analysis (*I* ^*2*^ = 63%, *p* = 0.04). The results showed that the experimental group was significantly better than the control group in improving SV in HFrEF patients (MD = 5.04, 95% CI: 3.67–6.40, *p* < 0.00001). There were four studies with a total of 328 patients mentioning CO (Gao et al., 2020; Su et al., 2020; Ma et al., 2021; Yao and Li, 2021). The heterogeneity suggests using the random effects model for analysis (*I* ^*2*^ = 96%, *p <* 0.00001). MD = 0.66, 95% CI: −0.12 to 1.43, *p <* 0.00001, means that the experimental group had no advantage in improving the CO of patients with HFrEF (Figure 10). According to the high heterogeneity of the CO, the literature analysis showed that the source of heterogeneity may be related to the disease severity of the patients.

### Adverse Events

**FIGURE 11:**
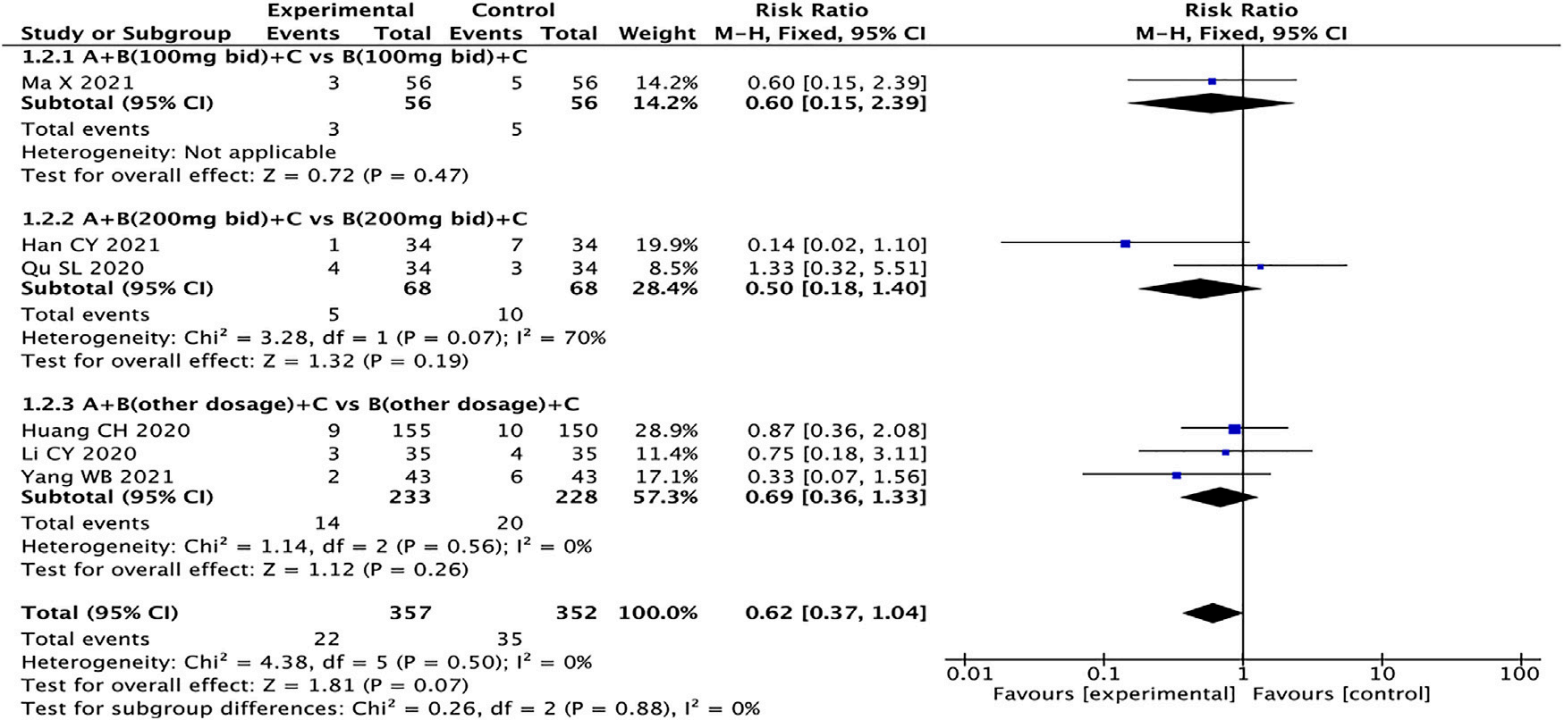
Forest plot of adverse events.

Six studies with a total of 709 patients reported adverse events (Huang et al., 2020; Li et al., 2020; Qu, 2020; Han, 2021; Ma et al., 2021; Yang, 2021), including dry cough (Li et al., 2020), dizziness (Li et al., 2020), headache (Li et al., 2020; Qu, 2020), hypotension (Han, 2021; Ma et al., 2021), palpitations (Li et al., 2020), angioedema (Huang et al., 2020; Li et al., 2020; Han, 2021; Ma et al., 2021), renal damage (Li et al., 2020; Han, 2021), abnormal serum potassium (Li et al., 2020; Han, 2021; Ma et al., 2021), and gastrointestinal adverse reactions such as nausea and vomiting (Huang et al., 2020; Li et al., 2020; Qu, 2020; Yang, 2021). The fixed effects model was used for statistical analysis (*I* ^*2*^ = 0%). The results showed that the experimental group had no advantage over the control group in reducing adverse events (RR = 0.62, 95% CI: 0.37–1.04, *p* = 0.07) (Figure 11).

### Evaluation of Publication Bias

**FIGURE 12:**
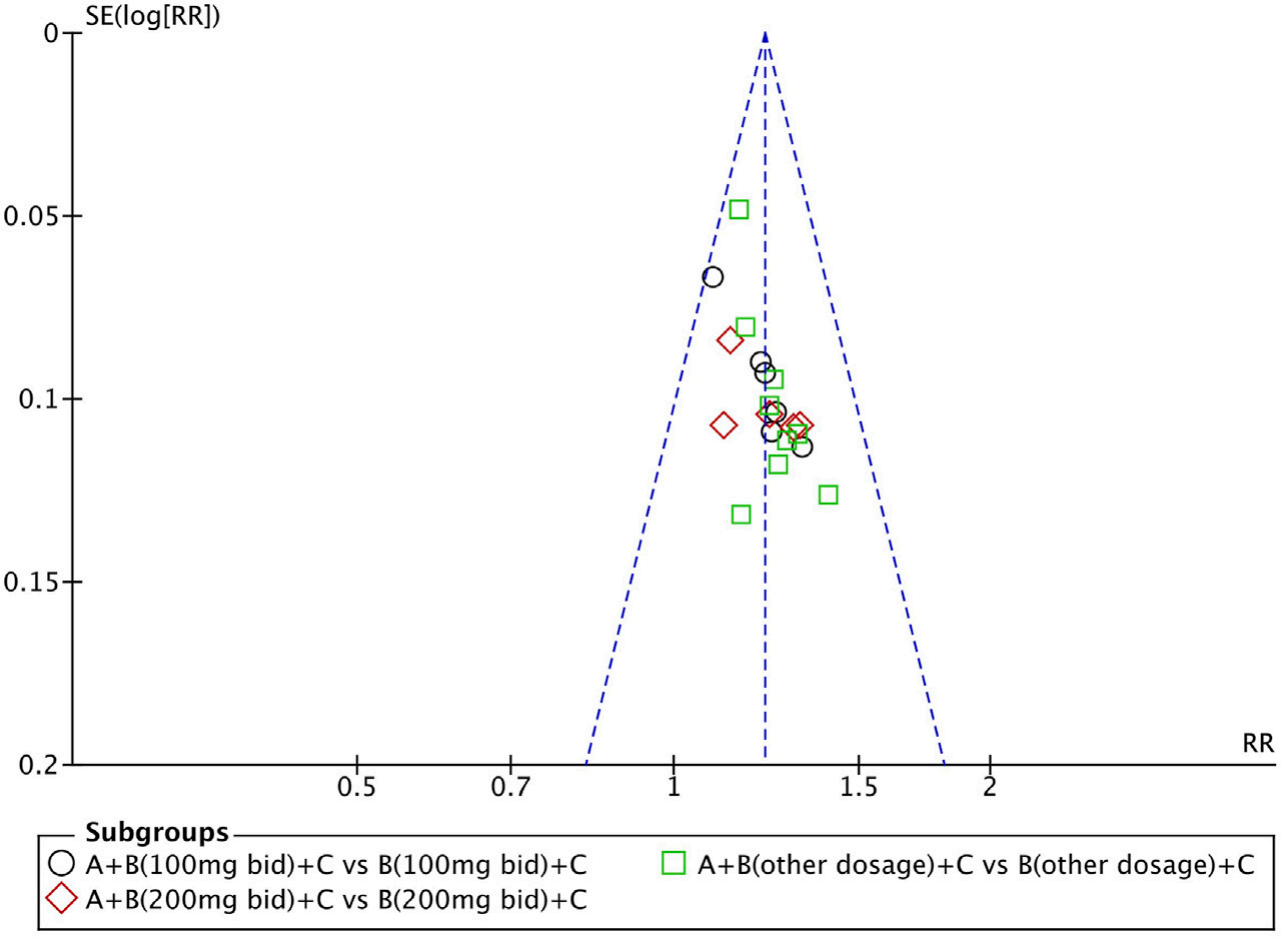
Funnel plot of the clinical efficacy rates: QQC combined with sacubitril/valsartan versus sacubitril/valsartan.

We used Review Manager Software 5.4 to evaluate publication bias based on the clinical efficacy rates. The funnel plot indicated that the studies were approximately evenly and symmetrically distributed within the inverted funnel plot, and two studies were slightly biased, suggesting possible publication bias (Figure 12). After excluding these two studies, the remaining studies were concentrated on both sides of the midline, and the distribution was symmetrical, indicating that the remaining studies may have less publication bias.

## Discussion

CHF is characterized by a progressive increase in cardiac filling pressure, decreased cardiac output, and decreased tissue oxygen delivery. These hemodynamic abnormalities lead to the activation of RAAS and the sympathetic nervous system to maintain blood perfusion of vital organs. In the early stage of the disease, this is an acute compensatory response, but continuous activation will cause a series of pathophysiological changes that aggravate the progression of HF, leading to abnormal heart and kidney function (Brewster et al., 2003).

ARNI represented by sacubitril/valsartan is an angiotensin receptor enkephalinase inhibitor. The study found that the application of sacubitril and valsartan can reduce myocardial fibrosis and left ventricular dilatation while delaying myocardial remodeling in HF patients (McMurray et al., 2013). Long-term use of ARNI can reduce the inner diameter of the left atrium and lower the volume index of the left atrium (Li et al., 2021).

Modern pharmacological studies have shown that the core active ingredients of QQC, ginsenoside-Rb2, palmitic acid, astragaloside IV, salvianolic acid A, salvianolic acid B, aconitine, hypoaconitine (Zhang et al., 2019), and so forth can treat HF by the following mechanisms. First, QQC can inhibit oxidative stress-induced cardiomyocyte apoptosis. For example, palmitic acid has been shown to reduce the expression level of Bcl-2 and activate the MAPK signaling pathway to achieve anti-apoptotic effects. Ginsenosides can enhance myocardial contractility, maintain myocardial cell integrity, and inhibit myocardial cell apoptosis (Liu, 2014; Liu et al., 2017). It can also regulate PI3K/AKT/GSK3*β* signaling pathway, reduce Bax and cytochrome c, and make Apaf-1, cleaved caspase-9, and cleaved caspase-3 express apoptosis, upregulate phosphorylase ratio, and improve oxidative stress-induced cardiomyocytes mitochondrial-dependent apoptosis (Zhang et al., 2016; Zhao et al., 2019). Secondly, QQC has a targeted role in regulating myocardial metabolism. It can simultaneously regulate myocardial glucose and FA metabolism through enzyme metabolism such as GLUT4 and GLUT1 (Shen et al., 2017; Wang et al., 2017), enhance glucose uptake in remote areas of the heart, and promote the conversion of the glucose oxidation pathway to a glycolysis solution. In addition, astragaloside IV can also increase the energy supply of fatty acid *β*-oxidation in cardiomyocytes by activating PPAR*α* (Dong et al., 2017) to maintain cell metabolism under hypoxia and low glucose conditions. On the contrary, for cardiomyocytes with better perfusion, the glucose oxidation pathway is uninhibited, which helps reduce the accumulation of metabolites and lactic acid produced by glycolysis (Cheng et al., 2020). Finally, QQC has outstanding performance in enhancing myocardial contractility and reducing ventricular remodeling. It can enhance the viability of cardiomyocytes by preventing the influx of calcium ions (Ma et al., 2013). Moreover, QQC can block the biological effect of TGF-*β*1 and inhibit the intermyocardial fibrosis, delaying or inhibiting ventricular remodeling (Chen et al., 2019).

Through the statistical analysis of a total of 2,427 patients in the 26 included studies, QQC combined with sacubitril/valsartan has advantages in the treatment of HFrEF, which is reflected in the total clinical effective rate, the improvement of 6-MWT, LVEF, LVEDD, LVESD, NT-proBNP, BNP, SV, AngII and ALD, and the difference has statistical advantages. However, there is no advantage in improving CO and reducing the incidence of adverse events.

The results of this study confirmed the effectiveness and safety of QQC combined with sacubitril/valsartan, but there are also some shortcomings. For example, the quality of the included RCTs is generally not high. Only a few studies have detailed experimental procedures and methods, meaning this study is unable to compare the literature. In the included literature, only 10 studies used the randomization method, accounting for 38.46%, no studies mentioned allocation concealment method, and no studies mentioned the use of blinding methods and were judged as uncertain risk of bias. At the same time, the sample size of the included studies is generally small, with a total of 18 studies with a sample size of less than 100, accounting for 69%, which makes the results of the included studies unreliable. In the future, we look forward to incorporating more high-quality, large-sample data studies to provide more scientific and reliable data support for the clinical use of QQC combined with sacubitril/valsartan.

## Conclusion

In conclusion, QQC combined with sacubitril/valsartan is effective in treating HFrEF. In future studies, we hope to conduct more and larger RCTs and more objective observation indicators to evaluate the effect of treatment of HFrEF.

## Data Availability

The original contributions presented in the study are included in the article/Supplementary Material. Further inquiries can be directed to the corresponding author.

## References

[B1] Bozkurt B. Coats A. J. S. Tsutsui H. Abdelhamid C. M. Adamopoulos S. Albert N. (2021). Universal Definition and Classification of Heart Failure: a Report of the Heart Failure Society of America, Heart Failure Association of the European Society of Cardiology, Japanese Heart Failure Society and Writing Committee of the Universal Definition of Heart Failure. Eur. J. Heart Fail. 23 (3), 352–380. 10.1002/ejhf.2115 33605000

[B2] Bragazzi N. L. Zhong W. Shu J. Abu Much A. Lotan D. Grupper A. (2021). Burden of Heart Failure and Underlying Causes in 195 Countries and Territories from 1990 to 2017. Eur. J. Prev. Cardiol. 28, 1682–1690. 10.1093/eurjpc/zwaa147 33571994

[B3] Brewster U. C. Setaro J. F. Perazella M. A. (2003). The Renin-Angiotensin-Aldosterone System: Cardiorenal Effects and Implications for Renal and Cardiovascular Disease States. Am. J. Med. Sci. 326 (1), 15–24. 10.1097/00000441-200307000-00003 12861121

[B4] Chen H. Lou L. Zhang D. Zhao Y. Zhao J. Li C. (2019). Qiliqiangxin Capsule Improves Cardiac Function and Attenuates Cardiac Remodeling by Upregulating miR-133a after Myocardial Infarction in Rats. Evid. Based Complement. Alternat Med. 2019, 7528214. 10.1155/2019/7528214 31001355 PMC6437749

[B5] Cheng W. Wang L. Yang T. Wu A. Wang B. Li T. (2020). Qiliqiangxin Capsules Optimize Cardiac Metabolism Flexibility in Rats With Heart Failure After Myocardial Infarction. Front. Physiol. 11, 805. 10.3389/fphys.2020.00805 32848816 PMC7396640

[B6] Crea F. (2021). The Universal Definition of Heart Failure, Risk Prediction in Cardiogenic Shock, Artificial Intelligence in Cardiac Allograft Rejection, and the Genetics of Dilated Cardiomyopathy. Eur. Heart J. 42 (24), 2317–2320. 10.1093/eurheartj/ehab370 34153987

[B7] Dong Y. Du Q. -H. (2020). The Effect of Qili Qiangxin Capsule Combined with Sacubitril and Valsartan Sodium in the Treatment of Chronic Heart Failure and its Effect on the Level of NT-proBNP. Chin. Mod. Drug Appl. 14 (24), 153–155. 10.14164/j.cnki.cn11-5581/r.2020.24.070

[B8] Dong Z. Zhao P. Xu M. Zhang C. Guo W. Chen H. (2017). Astragaloside IV Alleviates Heart Failure via Activating PPAR*α* to Switch Glycolysis to Fatty Acid *β*-oxidation. Sci. Rep. 7, 2691. 10.1038/s41598-017-02360-5 28578382 PMC5457407

[B9] Gao Y. -Y. Liu F. Gao L. Wang H. -P. Liu H. L. (2020). Effects of Qili Qiangxin Capsules Combined with Sacubitril and Valsartan on Cardiac Function in Patients with Chronic Heart Failure. China Mod. Appl. Pharm. 37 (20), 2516–2520. 10.13748/j.cnki.issn1007-7693.2020.20.015

[B10] Han C. -Y. (2021). The Effect of Qili Qiangxin Capsule Combined with Sacubitril and Valsartan Sodium Tablets for Patients with Coronary Heart Disease Complicated by Heart Failure. Contemp. Med. J. 19 (20), 185–187. 10.3969/j.issn.2095-7629.2021.20.092

[B11] Hu S. S. Gao R. L. Liu L. S. (2019). Summary of "Chinese Cardiovascular Disease Report 2018. Chin. J. Circ. 34 (3), 209–220. 10.3969/j.issn.1000-3614.2019.03.001

[B12] Huang C. -H. Wang Y. -F. Wang S. -H. (2020). Comparison of the Effects of Different Combination Regimens of Qili Qiangxin Capsules in the Treatment of Chronic Heart Failure. Chin. J. Pract. Med. 47 (24), 115–117. 10.3760/cma.j.cn115689-20200909-04380

[B13] Li C. -Y. Zhang M. Zhao H. -B. Yuan J. -C. Sang J. -W. He Z. H. (2020). Observation on the Clinical Efficacy of Sacubatril and Valsartan Combined with Qili Qiangxin Capsules in the Treatment of Patients with Chronic Heart Failure. J. Difficult Difficult Dis. 19 (07), 667–671. 10.3969/j.issn.1671-6450.2020.07.006

[B14] Li Z. Chang F. -J. Li W. -B. (2021). Efficacy of Sacubatril-Valsartan in the Treatment of Elderly Patients with Congestive Heart Failure. J. Cardiovasc. Rehabil. Med. 30(05):548–552. 10.3969/j.issn.1008-0074.2021.05.10

[B15] Lin B. -B. Wang L. Ye J. (2021). Effects of Qili Qiangxin Capsules Combined with New ARNI on Plasma NT-proBNP, Ang-Ⅱ, ALD, Serum MMP-9 Levels and Cardiac Function in Patients with Chronic Heart Failure. Clin. Reasonable J. Medication 14 (13), 13–15. 10.15887/j.cnki.13-1389/r.2021.13.005

[B16] Liu J. (2020). The Effect of Qili Qiangxin Capsules Combined with Sacubitril and Valsartan on Motor Function and Cardiac Function in Patients with Chronic Heart Failure. World Latest Med. Inf. Abstr. (Continuous Electron. Journal) 20 (84), 165–166. 10.3969/j.issn.1671-3141.2020.84.081

[B17] Liu R. Li D. Li Y. (2017). Research Progress on the Pharmacological Effects of Ginsenosides. China Food Nutr. 23 (10), 68–72. 10.3969/j.issn.1006-9577.2017.10.016

[B18] Liu X. -M. (2014). The Protective Effect and Mechanism of Ginsenoside Rb3 and Rb2 Composition on Myocardial Ischemia-Reperfusion Injury in rats[D]. Changchun: Jilin University.

[B19] Ma L. -Y. Yin Y. -J. Zhang J. -F. (2016). Effects of Qili Qiangxin Capsule on the RAS System and Sympathetic Nervous System of the Hypothalamic Paraventricular Nucleus in Rats with Chronic Heart Failure. Chin. Pharmacol. Bull. 32 (04), 575–580. 10.3969/j.issn.1001-1978.2016.04.026

[B20] Ma X. Zhang K. Li H. Han S. Ma Z. Tu P. (2013). Extracts from Astragalus Membranaceus Limit Myocardial Cell Death and Improve Cardiac Function in a Rat Model of Myocardial Ischemia. J. Ethnopharmacol 149 (3), 720–728. 10.1016/j.jep.2013.07.036 23968862

[B21] Ma X. Fu B. Li Q. -X. Su Y. (2021). Effects of Qili Qiangxin Capsules Combined with Sacubitril and Valsartan Sodium Tablets in the Treatment of Chronic Heart Failure. Anhui Med. 42 (01), 62–66. 10.3969/j.issn.1000-0399.2021.01.015

[B22] McMurray J. J. Packer M. Desai A. S. Gong J. Lefkowitz M. P. Rizkala A. R. (2013). Dual Angiotensin Receptor and Neprilysin Inhibition as an Alternative to Angiotensin-Converting Enzyme Inhibition in Patients with Chronic Systolic Heart Failure: Rationale for and Design of the Prospective Comparison of ARNI with ACEI to Determine Impact on Global Mortality and Morbidity in Heart Failure Trial (PARADIGM-HF). Eur. J. Heart Fail. 15 (9), 1062–1073. 10.1093/eurjhf/hft052 23563576 PMC3746839

[B23] Qin S. -Q. Wang Y. -L. Liu J. -F. Zhao H. -T. Li Z. -R. (2020). Clinical Effects of Qili Qiangxin Capsule Combined with Sacubitril and Valsartan Sodium Tablets in the Treatment of Chronic Cardiac Insufficiency. Chin. J. Traditional Chin. Med. 38 (4), 201–203. 10.13193/j.issn.1673-7717.2020.04.047

[B24] Qu S. -L. (2020). Efficacy of Qili Qiangxin Capsules Combined with Sacubitril and Valsartan Sodium Tablets in the Treatment of Patients with Chronic Cardiac Insufficiency. Med. Equipment 33 (10), 81–82. 10.3969/j.issn.1002-2376.2020.10.050

[B25] Shen S. Jiang H. Bei Y. Zhang J. Zhang H. Zhu H. (2017). Qiliqiangxin Attenuates Adverse Cardiac Remodeling after Myocardial Infarction in Ovariectomized Mice via Activation of PPARγ. Cell Physiol. Biochem. 42, 876–888. 10.1159/000478641 28647730

[B26] Shi Y. -G. (2018). Efficacy of Qili Qiangxin Capsules Combined with Sacubitril and Valsartan Sodium Tablets in the Treatment of Chronic Heart Failure. China Contemp. Med. 25 (31), 56–58. 10.3969/j.issn.1674-4721.2018.31.019

[B27] Su C. -B. Xu J. -R. Chen P. -H. Lu D. Li S. -J. Tang Y. -P. (2020). Clinical Efficacy of Qili Qiangxin Capsule Combined with Sacubitril and Valsartan Sodium Tablets in the Treatment of Chronic Heart Failure and its Effect on Serum Matrix Lysin 2 Levels. Guangdong Med. Sci. J. Univ. 38 (01), 37–40. 10.3969/j.issn.1005-4057.2020.01.008

[B28] Su T. Sun B. -J. (2021). Efficacy of Sacubatril and Valsartan with Qili Qiangxin Capsules in the Treatment of Dilated Cardiomyopathy and Heart Failure. Self-care. 6 (13), 40.

[B29] Tan G. -C. (2021). The Application Effect of Sacubitril and Valsartan Combined with Qili Qiangxin Capsule in the Treatment of Heart Failure. Famous Doctor 4 (08), 153–154.

[B30] Teerlink J. R. Voors A. A. Ponikowski P. Pang P. S. Greenberg B. H. Filippatos G. (2017). Serelaxin in Addition to Standard Therapy in Acute Heart Failure: Rationale and Design of the RELAX-AHF-2 Study. Eur. J. Heart Fail. 19 (6), 800–809. 10.1002/ejhf.830 28452195 PMC5488179

[B31] Toischer K. Rokita A. G. Unsöld B. Zhu W. Kararigas G. Sossalla S. (2010). Differential Cardiac Remodeling in Preload versus Afterload. Circulation 122 (10), 993–1003. 10.1161/CIRCULATIONAHA.110.943431 20733099 PMC2955196

[B32] Wang H. -L. Shang B. B. (2021). The Clinical Study of Sacubitril and Valsartan Combined with Qili Qiangxin Capsule in the Treatment of Dilated Cardiomyopathy and Heart Failure. Kang Yi 11 (16), 218. 10.12332/j.issn.2095-6525.2021.16.209

[B33] Wang H. -Y. Xu T. Zhang H. -P. (2021). The Application of Sacubatril and Valsartan Sodium Tablets Combined with Qili Qiangxin Capsules in the Treatment of Chronic Heart Failure. J. Hebei North Univ. (Natural Sci. Edition) 37 (04), 42–43. 10.3969/j.issn.1673-1492.2021.04.011

[B34] Wang H. Liang Y. -C. (2018). Chinese Guidelines for the Diagnosis and Treatment of Heart Failure 2018. Chin. J. Cardiovasc. Dis. 46 (10), 760–789. 10.12102/j.issn.1672-1349.2020.15.020 30369168

[B35] Wang J. Li Z. Wang Y. Zhang J. Zhao W. Fu M. (2017). Qiliqiangxin Enhances Cardiac Glucose Metabolism and Improves Diastolic Function in Spontaneously Hypertensive Rats. Evid. Based Complement. Alternat. Med. 2017, 3197320. 10.1155/2017/3197320 28706558 PMC5494577

[B36] Wang L. (2019). Clinical Effect Observation of Qili Qiangxin Capsule Combined with Sacubitril and Valsartan Sodium Tablets in the Treatment of Chronic Heart Failure. J. Clin. Rational Use 12 (29), 3–4. 10.15887/j.cnki.13-1389/r.2019.29.002

[B37] Wang Q. (2021). Study on the Clinical Effect of Qili Qiangxin Capsules Combined with Sacubatril and Valsartan in the Treatment of Chronic Heart Failure. Heilongjiang Traditional Chin. Med. 50 (02), 44–45.

[B38] Wang S. -K. Zhang Z. Wang C. -M. Zhou M. Cheng L. -D. Peng H. (2020). Clinical Study of Sacubatril and Valsartan Combined with Qili Qiangxin Capsules in the Treatment of Dilated Cardiomyopathy and Heart Failure. J. Integrated Traditional Chin. West. Med. Cardio-Cerebrovascular Dis. 18 (21), 3620–3622. 10.12102/j.issn.1672-1349.2020.21.027

[B39] Xu J. -F. (2020). Clinical Study on Qili Qiangxin Capsule Combined with Sacubitril and Valsartan Sodium Tablets in the Treatment of Chronic Heart Failure. J. Pract. Traditional Chin. Med. 36 (04), 507–509.

[B40] Yang W. -B. (2021). The Effect of Qili Qiangxin Capsules Combined with Sacubatril and Valsartan Sodium on Chronic Heart Failure. Pract. Clin. Med. Integrated Traditional Chin. West. Med. 21 (14), 66–67. 10.13638/j.issn.1671-4040.2021.14.029

[B41] Yao Y. Li C. -X. (2021). Effect Analysis of Qili Qiangxin Capsule Combined with Sacubitril and Valsartan Sodium Tablets in the Treatment of Chronic Heart Failure. Health Vis. 2021 (19), 107.

[B42] Zhang C. -H. (2020). Efficacy Analysis of Qili Qiangxin Capsule Combined with Sacubitril and Valsartan Sodium Tablets in the Treatment of Chronic Heart Failure. China Mod. Med. Appl. 14 (01), 129–131. 10.14164/j.cnki.cn11-5581/r.2020.01.067

[B43] Zhang L. P. Jiang Y. C. Yu X. F. Xu H. L. Li M. Zhao X. Z. (2016). Ginsenoside Rg3 Improves Cardiac Function after Myocardial Ischemia/reperfusion via Attenuating Apoptosis and Inflammation. Evid. Based Complement. Alternat Med. 2016, 6967853. 10.1155/2016/6967853 28105061 PMC5220470

[B44] Zhang Y. Zhu M. Zhang F. Zhang S. Du W. Xiao X. (2019). Integrating Pharmacokinetics Study, Network Analysis, and Experimental Validation to Uncover the Mechanism of Qiliqiangxin Capsule Against Chronic Heart Failure. Front. Pharmacol. 10 (10), 1046. 10.3389/fphar.2019.01046 31619994 PMC6759796

[B45] Zhang Y. -P. (2021). The Application Value of Qili Qiangxin Capsule Combined with Western Medicine in Patients with Chronic Heart Failure. Henan Med. Res. 30 (05), 927–929. 10.3969/j.issn.1004-437X.2021.05.063

[B46] Zhao Q. L. -H. Li H. Chang L. Wei C. Yin Y. Bei H. (2019). Qiliqiangxin Attenuates Oxidative Stress-Induced Mitochondrion-dependent Apoptosis in Cardiomyocytes via PI3K/AKT/GSK3*β* Signaling Pathway. Biol. Pharm. Bull. 42 (8), 1310–1321. Epub 2019 May 28. PMID: 31142701. 10.1248/bpb.b19-00050 31142701

[B47] Zhao Y. -Q. Chang X. -H. Cui W. -Y. Liu H. Yang Y. -N. (2020a). Curative Effect of Qili Qiangxin Capsule Combined with Sacubitril and Valsartan in the Treatment of Chronic Heart Failure with Atrial Fibrillation and its Effect on Cardiac Function. Mod. J. Integrated Traditional Chin. West. Med. 29 (35), 3939–3943. 10.3969/j.issn.1008-8849.2020.35.015

[B48] Zhao Y. -Q. Liu H. Yang Y. -N. Wang L. Qiao L. -N. Liu X. -Y. (2020b). Observation on the Efficacy of Qili Qiangxin Capsule Combined with Sacubitril and Valsartan in the Treatment of Heart Failure with Atrial Fibrillation. J. Integrated Traditional Chin. West. Med. Cardiovasc. Cerebrovasc. Dis. 18 (15), 2457–2459. 10.12102/j.issn.1672-1349.2020.15.020

