# Supplementary material for: Qili Qiangxin Capsule Combined With Sacubitril/Valsartan for HFrEF: A Systematic Review and Meta-Analysis
Source: Front Pharmacol. 2022 Apr 4;13:832782. doi: 10.3389/fphar.2022.832782 (PMC9014182; doi:10.3389/fphar.2022.832782)
Supplement: Supplementary file 1 [file Table1.DOCX]

| **SUMMARY TABLE OF THE STUDIES INCLUDED**. | | | | | |
| --- | --- | --- | --- | --- | --- |
| **Study** | **Formulation** | **Source** | **Species, concentration** | **Quality control reported**  **(Y/N)** | **Chemical analysis reported**  **(Y/N)** |
| Dong Y  2020 | Qili Qiangxin Capsule | Shijiazhuang Yiling Pharmaceutical Co., Ltd. | - Root of *Astragalus mongholicus Bunge.* [Membranaceus Bunge; Astragali Radix.] - concentration uncertainty - Root of *Panax ginseng C.A.Mey*. [Araliaceae; Ginseng Radix Et Rhizoma]   concentration uncertainty   - Root of *Aconitum carmichaelii Debx*. [Ranunculaceae; Aconiti Lateralis Radix Praeparata]   concentration uncertainty   - Root of *Salvia miltiorrhiza Bunge*. [Labiatae; Salviae Miltiorrhizae]   concentration uncertainty   - Mature seeds of *Descurainia sophia (L.) Webb ex Prantl.* [Cruciferae; Descurainiae Semen]   concentration uncertainty   - Tuber of *Alisma plantago-aquatica subsp. orientale (Sam.) Sam.* [Alisaceae; oriental waterplantain rhizome]   concentration uncertainty   - Dry rhizome of *Polygonatum odoratum (Mill.) Druce*. [Liliaceae; Polygonatum]   concentration uncertainty   - Twigs of *Neolitsea cassia (L.) Kosterm.* [Lauraceae; Cinnamomi Ramulus]   concentration uncertainty   - Flower of *Carthamus tinctorius L*. [Compositae; Carthami Flos]   concentration uncertainty   - Root bark of *Periploca sepium Bunge.* [Asclepiaceae; Cortex Periplocae]   concentration uncertainty   - The ripe peel of T*angerine and its cultivars*. [Rutaceae; Citrus × aurantium L.]   concentration uncertainty | Y – Prepared according to the Pharmacopoeia of China, 2020 edition | N |
| Gao YY  2020 | Qili Qiangxin Capsule | Shijiazhuang Yiling Pharmaceutical Co., Ltd. | - Root of *Astragalus mongholicus Bunge.* [Membranaceus Bunge; Astragali Radix.] - concentration uncertainty - Root of *Panax ginseng C.A.Mey*. [Araliaceae; Ginseng Radix Et Rhizoma]   concentration uncertainty   - Root of *Aconitum carmichaelii Debx*. [Ranunculaceae; Aconiti Lateralis Radix Praeparata]   concentration uncertainty   - Root of *Salvia miltiorrhiza Bunge*. [Labiatae; Salviae Miltiorrhizae]   concentration uncertainty   - Mature seeds of *Descurainia sophia (L.) Webb ex Prantl.* [Cruciferae; Descurainiae Semen]   concentration uncertainty   - Tuber of *Alisma plantago-aquatica subsp. orientale (Sam.) Sam.* [Alisaceae; oriental waterplantain rhizome]   concentration uncertainty   - Dry rhizome of *Polygonatum odoratum (Mill.) Druce*. [Liliaceae; Polygonatum]   concentration uncertainty   - Twigs of *Neolitsea cassia (L.) Kosterm.* [Lauraceae; Cinnamomi Ramulus]   concentration uncertainty   - Flower of *Carthamus tinctorius L*. [Compositae; Carthami Flos]   concentration uncertainty   - Root bark of *Periploca sepium Bunge.* [Asclepiaceae; Cortex Periplocae]   concentration uncertainty   - The ripe peel of T*angerine and its cultivars*. [Rutaceae; Citrus × aurantium L.]   concentration uncertainty | Y – Prepared according to the Pharmacopoeia of China, 2020 edition | N |
| Hang CY  2021 | Qili Qiangxin Capsule | Shijiazhuang Yiling Pharmaceutical Co., Ltd. | - Root of *Astragalus mongholicus Bunge.* [Membranaceus Bunge; Astragali Radix.] - concentration uncertainty - Root of *Panax ginseng C.A.Mey*. [Araliaceae; Ginseng Radix Et Rhizoma]   concentration uncertainty   - Root of *Aconitum carmichaelii Debx*. [Ranunculaceae; Aconiti Lateralis Radix Praeparata]   concentration uncertainty   - Root of *Salvia miltiorrhiza Bunge*. [Labiatae; Salviae Miltiorrhizae]   concentration uncertainty   - Mature seeds of *Descurainia sophia (L.) Webb ex Prantl.* [Cruciferae; Descurainiae Semen]   concentration uncertainty   - Tuber of *Alisma plantago-aquatica subsp. orientale (Sam.) Sam.* [Alisaceae; oriental waterplantain rhizome]   concentration uncertainty   - Dry rhizome of *Polygonatum odoratum (Mill.) Druce*. [Liliaceae; Polygonatum]   concentration uncertainty   - Twigs of *Neolitsea cassia (L.) Kosterm.* [Lauraceae; Cinnamomi Ramulus]   concentration uncertainty   - Flower of *Carthamus tinctorius L*. [Compositae; Carthami Flos]   concentration uncertainty   - Root bark of *Periploca sepium Bunge.* [Asclepiaceae; Cortex Periplocae]   concentration uncertainty   - The ripe peel of T*angerine and its cultivars*. [Rutaceae; Citrus × aurantium L.]   concentration uncertainty | Y – Prepared according to the Pharmacopoeia of China, 2020 edition | N |
| Huang CH  2020 | Qili Qiangxin Capsule | Shijiazhuang Yiling Pharmaceutical Co., Ltd. | - Root of *Astragalus mongholicus Bunge.* [Membranaceus Bunge; Astragali Radix.] - concentration uncertainty - Root of *Panax ginseng C.A.Mey*. [Araliaceae; Ginseng Radix Et Rhizoma]   concentration uncertainty   - Root of *Aconitum carmichaelii Debx*. [Ranunculaceae; Aconiti Lateralis Radix Praeparata]   concentration uncertainty   - Root of *Salvia miltiorrhiza Bunge*. [Labiatae; Salviae Miltiorrhizae]   concentration uncertainty   - Mature seeds of *Descurainia sophia (L.) Webb ex Prantl.* [Cruciferae; Descurainiae Semen]   concentration uncertainty   - Tuber of *Alisma plantago-aquatica subsp. orientale (Sam.) Sam.* [Alisaceae; oriental waterplantain rhizome]   concentration uncertainty   - Dry rhizome of *Polygonatum odoratum (Mill.) Druce*. [Liliaceae; Polygonatum]   concentration uncertainty   - Twigs of *Neolitsea cassia (L.) Kosterm.* [Lauraceae; Cinnamomi Ramulus]   concentration uncertainty   - Flower of *Carthamus tinctorius L*. [Compositae; Carthami Flos]   concentration uncertainty   - Root bark of *Periploca sepium Bunge.* [Asclepiaceae; Cortex Periplocae]   concentration uncertainty   - The ripe peel of T*angerine and its cultivars*. [Rutaceae; Citrus × aurantium L.]   concentration uncertainty | Y – Prepared according to the Pharmacopoeia of China, 2020 edition | N |
| Li CY  2020 | Qili Qiangxin Capsule | Shijiazhuang Yiling Pharmaceutical Co., Ltd. | - Root of *Astragalus mongholicus Bunge.* [Membranaceus Bunge; Astragali Radix.] - concentration uncertainty - Root of *Panax ginseng C.A.Mey*. [Araliaceae; Ginseng Radix Et Rhizoma]   concentration uncertainty   - Root of *Aconitum carmichaelii Debx*. [Ranunculaceae; Aconiti Lateralis Radix Praeparata]   concentration uncertainty   - Root of *Salvia miltiorrhiza Bunge*. [Labiatae; Salviae Miltiorrhizae]   concentration uncertainty   - Mature seeds of *Descurainia sophia (L.) Webb ex Prantl.* [Cruciferae; Descurainiae Semen]   concentration uncertainty   - Tuber of *Alisma plantago-aquatica subsp. orientale (Sam.) Sam.* [Alisaceae; oriental waterplantain rhizome]   concentration uncertainty   - Dry rhizome of *Polygonatum odoratum (Mill.) Druce*. [Liliaceae; Polygonatum]   concentration uncertainty   - Twigs of *Neolitsea cassia (L.) Kosterm.* [Lauraceae; Cinnamomi Ramulus]   concentration uncertainty   - Flower of *Carthamus tinctorius L*. [Compositae; Carthami Flos]   concentration uncertainty   - Root bark of *Periploca sepium Bunge.* [Asclepiaceae; Cortex Periplocae]   concentration uncertainty   - The ripe peel of T*angerine and its cultivars*. [Rutaceae; Citrus × aurantium L.]   concentration uncertainty | Y – Prepared according to the Pharmacopoeia of China, 2020 edition | N |
| Lin BB  2021 | Qili Qiangxin Capsule | Shijiazhuang Yiling Pharmaceutical Co., Ltd. | - Root of *Astragalus mongholicus Bunge.* [Membranaceus Bunge; Astragali Radix.] - concentration uncertainty - Root of *Panax ginseng C.A.Mey*. [Araliaceae; Ginseng Radix Et Rhizoma]   concentration uncertainty   - Root of *Aconitum carmichaelii Debx*. [Ranunculaceae; Aconiti Lateralis Radix Praeparata]   concentration uncertainty   - Root of *Salvia miltiorrhiza Bunge*. [Labiatae; Salviae Miltiorrhizae]   concentration uncertainty   - Mature seeds of *Descurainia sophia (L.) Webb ex Prantl.* [Cruciferae; Descurainiae Semen]   concentration uncertainty   - Tuber of *Alisma plantago-aquatica subsp. orientale (Sam.) Sam.* [Alisaceae; oriental waterplantain rhizome]   concentration uncertainty   - Dry rhizome of *Polygonatum odoratum (Mill.) Druce*. [Liliaceae; Polygonatum]   concentration uncertainty   - Twigs of *Neolitsea cassia (L.) Kosterm.* [Lauraceae; Cinnamomi Ramulus]   concentration uncertainty   - Flower of *Carthamus tinctorius L*. [Compositae; Carthami Flos]   concentration uncertainty   - Root bark of *Periploca sepium Bunge.* [Asclepiaceae; Cortex Periplocae]   concentration uncertainty   - The ripe peel of T*angerine and its cultivars*. [Rutaceae; Citrus × aurantium L.]   concentration uncertainty | Y – Prepared according to the Pharmacopoeia of China, 2020 edition | N |
| Liu J  2020 | Qili Qiangxin Capsule | Shijiazhuang Yiling Pharmaceutical Co., Ltd. | - Root of *Astragalus mongholicus Bunge.* [Membranaceus Bunge; Astragali Radix.] - concentration uncertainty - Root of *Panax ginseng C.A.Mey*. [Araliaceae; Ginseng Radix Et Rhizoma]   concentration uncertainty   - Root of *Aconitum carmichaelii Debx*. [Ranunculaceae; Aconiti Lateralis Radix Praeparata]   concentration uncertainty   - Root of *Salvia miltiorrhiza Bunge*. [Labiatae; Salviae Miltiorrhizae]   concentration uncertainty   - Mature seeds of *Descurainia sophia (L.) Webb ex Prantl.* [Cruciferae; Descurainiae Semen]   concentration uncertainty   - Tuber of *Alisma plantago-aquatica subsp. orientale (Sam.) Sam.* [Alisaceae; oriental waterplantain rhizome]   concentration uncertainty   - Dry rhizome of *Polygonatum odoratum (Mill.) Druce*. [Liliaceae; Polygonatum]   concentration uncertainty   - Twigs of *Neolitsea cassia (L.) Kosterm.* [Lauraceae; Cinnamomi Ramulus]   concentration uncertainty   - Flower of *Carthamus tinctorius L*. [Compositae; Carthami Flos]   concentration uncertainty   - Root bark of *Periploca sepium Bunge.* [Asclepiaceae; Cortex Periplocae]   concentration uncertainty   - The ripe peel of T*angerine and its cultivars*. [Rutaceae; Citrus × aurantium L.]   concentration uncertainty | Y – Prepared according to the Pharmacopoeia of China, 2020 edition | N |
| Ma X  2021 | Qili Qiangxin Capsule | Shijiazhuang Yiling Pharmaceutical Co., Ltd. | - Root of *Astragalus mongholicus Bunge.* [Membranaceus Bunge; Astragali Radix.] - concentration uncertainty - Root of *Panax ginseng C.A.Mey*. [Araliaceae; Ginseng Radix Et Rhizoma]   concentration uncertainty   - Root of *Aconitum carmichaelii Debx*. [Ranunculaceae; Aconiti Lateralis Radix Praeparata]   concentration uncertainty   - Root of *Salvia miltiorrhiza Bunge*. [Labiatae; Salviae Miltiorrhizae]   concentration uncertainty   - Mature seeds of *Descurainia sophia (L.) Webb ex Prantl.* [Cruciferae; Descurainiae Semen]   concentration uncertainty   - Tuber of *Alisma plantago-aquatica subsp. orientale (Sam.) Sam.* [Alisaceae; oriental waterplantain rhizome]   concentration uncertainty   - Dry rhizome of *Polygonatum odoratum (Mill.) Druce*. [Liliaceae; Polygonatum]   concentration uncertainty   - Twigs of *Neolitsea cassia (L.) Kosterm.* [Lauraceae; Cinnamomi Ramulus]   concentration uncertainty   - Flower of *Carthamus tinctorius L*. [Compositae; Carthami Flos]   concentration uncertainty   - Root bark of *Periploca sepium Bunge.* [Asclepiaceae; Cortex Periplocae]   concentration uncertainty   - The ripe peel of T*angerine and its cultivars*. [Rutaceae; Citrus × aurantium L.]   concentration uncertainty | Y – Prepared according to the Pharmacopoeia of China, 2020 edition | N |
| Qin SQ  2020 | Qili Qiangxin Capsule | Shijiazhuang Yiling Pharmaceutical Co., Ltd. | - Root of *Astragalus mongholicus Bunge.* [Membranaceus Bunge; Astragali Radix.] - concentration uncertainty - Root of *Panax ginseng C.A.Mey*. [Araliaceae; Ginseng Radix Et Rhizoma]   concentration uncertainty   - Root of *Aconitum carmichaelii Debx*. [Ranunculaceae; Aconiti Lateralis Radix Praeparata]   concentration uncertainty   - Root of *Salvia miltiorrhiza Bunge*. [Labiatae; Salviae Miltiorrhizae]   concentration uncertainty   - Mature seeds of *Descurainia sophia (L.) Webb ex Prantl.* [Cruciferae; Descurainiae Semen]   concentration uncertainty   - Tuber of *Alisma plantago-aquatica subsp. orientale (Sam.) Sam.* [Alisaceae; oriental waterplantain rhizome]   concentration uncertainty   - Dry rhizome of *Polygonatum odoratum (Mill.) Druce*. [Liliaceae; Polygonatum]   concentration uncertainty   - Twigs of *Neolitsea cassia (L.) Kosterm.* [Lauraceae; Cinnamomi Ramulus]   concentration uncertainty   - Flower of *Carthamus tinctorius L*. [Compositae; Carthami Flos]   concentration uncertainty   - Root bark of *Periploca sepium Bunge.* [Asclepiaceae; Cortex Periplocae]   concentration uncertainty   - The ripe peel of T*angerine and its cultivars*. [Rutaceae; Citrus × aurantium L.]   concentration uncertainty | Y – Prepared according to the Pharmacopoeia of China, 2020 edition | N |
| Qu SL  2020 | Qili Qiangxin Capsule | Shijiazhuang Yiling Pharmaceutical Co., Ltd. | - Root of *Astragalus mongholicus Bunge.* [Membranaceus Bunge; Astragali Radix.] - concentration uncertainty - Root of *Panax ginseng C.A.Mey*. [Araliaceae; Ginseng Radix Et Rhizoma]   concentration uncertainty   - Root of *Aconitum carmichaelii Debx*. [Ranunculaceae; Aconiti Lateralis Radix Praeparata]   concentration uncertainty   - Root of *Salvia miltiorrhiza Bunge*. [Labiatae; Salviae Miltiorrhizae]   concentration uncertainty   - Mature seeds of *Descurainia sophia (L.) Webb ex Prantl.* [Cruciferae; Descurainiae Semen]   concentration uncertainty   - Tuber of *Alisma plantago-aquatica subsp. orientale (Sam.) Sam.* [Alisaceae; oriental waterplantain rhizome]   concentration uncertainty   - Dry rhizome of *Polygonatum odoratum (Mill.) Druce*. [Liliaceae; Polygonatum]   concentration uncertainty   - Twigs of *Neolitsea cassia (L.) Kosterm.* [Lauraceae; Cinnamomi Ramulus]   concentration uncertainty   - Flower of *Carthamus tinctorius L*. [Compositae; Carthami Flos]   concentration uncertainty   - Root bark of *Periploca sepium Bunge.* [Asclepiaceae; Cortex Periplocae]   concentration uncertainty   - The ripe peel of T*angerine and its cultivars*. [Rutaceae; Citrus × aurantium L.]   concentration uncertainty | Y – Prepared according to the Pharmacopoeia of China, 2020 edition | N |
| Shi YG  2018 | Qili Qiangxin Capsule | Shijiazhuang Yiling Pharmaceutical Co., Ltd. | - Root of *Astragalus mongholicus Bunge.* [Membranaceus Bunge; Astragali Radix.] - concentration uncertainty - Root of *Panax ginseng C.A.Mey*. [Araliaceae; Ginseng Radix Et Rhizoma]   concentration uncertainty   - Root of *Aconitum carmichaelii Debx*. [Ranunculaceae; Aconiti Lateralis Radix Praeparata]   concentration uncertainty   - Root of *Salvia miltiorrhiza Bunge*. [Labiatae; Salviae Miltiorrhizae]   concentration uncertainty   - Mature seeds of *Descurainia sophia (L.) Webb ex Prantl.* [Cruciferae; Descurainiae Semen]   concentration uncertainty   - Tuber of *Alisma plantago-aquatica subsp. orientale (Sam.) Sam.* [Alisaceae; oriental waterplantain rhizome]   concentration uncertainty   - Dry rhizome of *Polygonatum odoratum (Mill.) Druce*. [Liliaceae; Polygonatum]   concentration uncertainty   - Twigs of *Neolitsea cassia (L.) Kosterm.* [Lauraceae; Cinnamomi Ramulus]   concentration uncertainty   - Flower of *Carthamus tinctorius L*. [Compositae; Carthami Flos]   concentration uncertainty   - Root bark of *Periploca sepium Bunge.* [Asclepiaceae; Cortex Periplocae]   concentration uncertainty   - The ripe peel of T*angerine and its cultivars*. [Rutaceae; Citrus × aurantium L.]   concentration uncertainty | Y – Prepared according to the Pharmacopoeia of China, 2020 edition | N |
| Su CB  2020 | Qili Qiangxin Capsule | Shijiazhuang Yiling Pharmaceutical Co., Ltd. | - Root of *Astragalus mongholicus Bunge.* [Membranaceus Bunge; Astragali Radix.] - concentration uncertainty - Root of *Panax ginseng C.A.Mey*. [Araliaceae; Ginseng Radix Et Rhizoma]   concentration uncertainty   - Root of *Aconitum carmichaelii Debx*. [Ranunculaceae; Aconiti Lateralis Radix Praeparata]   concentration uncertainty   - Root of *Salvia miltiorrhiza Bunge*. [Labiatae; Salviae Miltiorrhizae]   concentration uncertainty   - Mature seeds of *Descurainia sophia (L.) Webb ex Prantl.* [Cruciferae; Descurainiae Semen]   concentration uncertainty   - Tuber of *Alisma plantago-aquatica subsp. orientale (Sam.) Sam.* [Alisaceae; oriental waterplantain rhizome]   concentration uncertainty   - Dry rhizome of *Polygonatum odoratum (Mill.) Druce*. [Liliaceae; Polygonatum]   concentration uncertainty   - Twigs of *Neolitsea cassia (L.) Kosterm.* [Lauraceae; Cinnamomi Ramulus]   concentration uncertainty   - Flower of *Carthamus tinctorius L*. [Compositae; Carthami Flos]   concentration uncertainty   - Root bark of *Periploca sepium Bunge.* [Asclepiaceae; Cortex Periplocae]   concentration uncertainty   - The ripe peel of T*angerine and its cultivars*. [Rutaceae; Citrus × aurantium L.]   concentration uncertainty | Y – Prepared according to the Pharmacopoeia of China, 2020 edition | N |
| Su T  2021 | Qili Qiangxin Capsule | Shijiazhuang Yiling Pharmaceutical Co., Ltd. | - Root of *Astragalus mongholicus Bunge.* [Membranaceus Bunge; Astragali Radix.] - concentration uncertainty - Root of *Panax ginseng C.A.Mey*. [Araliaceae; Ginseng Radix Et Rhizoma]   concentration uncertainty   - Root of *Aconitum carmichaelii Debx*. [Ranunculaceae; Aconiti Lateralis Radix Praeparata]   concentration uncertainty   - Root of *Salvia miltiorrhiza Bunge*. [Labiatae; Salviae Miltiorrhizae]   concentration uncertainty   - Mature seeds of *Descurainia sophia (L.) Webb ex Prantl.* [Cruciferae; Descurainiae Semen]   concentration uncertainty   - Tuber of *Alisma plantago-aquatica subsp. orientale (Sam.) Sam.* [Alisaceae; oriental waterplantain rhizome]   concentration uncertainty   - Dry rhizome of *Polygonatum odoratum (Mill.) Druce*. [Liliaceae; Polygonatum]   concentration uncertainty   - Twigs of *Neolitsea cassia (L.) Kosterm.* [Lauraceae; Cinnamomi Ramulus]   concentration uncertainty   - Flower of *Carthamus tinctorius L*. [Compositae; Carthami Flos]   concentration uncertainty   - Root bark of *Periploca sepium Bunge.* [Asclepiaceae; Cortex Periplocae]   concentration uncertainty   - The ripe peel of T*angerine and its cultivars*. [Rutaceae; Citrus × aurantium L.]   concentration uncertainty | Y – Prepared according to the Pharmacopoeia of China, 2020 edition | N |
| Tan GC  2021 | Qili Qiangxin Capsule | Shijiazhuang Yiling Pharmaceutical Co., Ltd. | - Root of *Astragalus mongholicus Bunge.* [Membranaceus Bunge; Astragali Radix.] - concentration uncertainty - Root of *Panax ginseng C.A.Mey*. [Araliaceae; Ginseng Radix Et Rhizoma]   concentration uncertainty   - Root of *Aconitum carmichaelii Debx*. [Ranunculaceae; Aconiti Lateralis Radix Praeparata]   concentration uncertainty   - Root of *Salvia miltiorrhiza Bunge*. [Labiatae; Salviae Miltiorrhizae]   concentration uncertainty   - Mature seeds of *Descurainia sophia (L.) Webb ex Prantl.* [Cruciferae; Descurainiae Semen]   concentration uncertainty   - Tuber of *Alisma plantago-aquatica subsp. orientale (Sam.) Sam.* [Alisaceae; oriental waterplantain rhizome]   concentration uncertainty   - Dry rhizome of *Polygonatum odoratum (Mill.) Druce*. [Liliaceae; Polygonatum]   concentration uncertainty   - Twigs of *Neolitsea cassia (L.) Kosterm.* [Lauraceae; Cinnamomi Ramulus]   concentration uncertainty   - Flower of *Carthamus tinctorius L*. [Compositae; Carthami Flos]   concentration uncertainty   - Root bark of *Periploca sepium Bunge.* [Asclepiaceae; Cortex Periplocae]   concentration uncertainty   - The ripe peel of T*angerine and its cultivars*. [Rutaceae; Citrus × aurantium L.]   concentration uncertainty | Y – Prepared according to the Pharmacopoeia of China, 2020 edition | N |
| Wang HL  2021 | Qili Qiangxin Capsule | Shijiazhuang Yiling Pharmaceutical Co., Ltd. | - Root of *Astragalus mongholicus Bunge.* [Membranaceus Bunge; Astragali Radix.] - concentration uncertainty - Root of *Panax ginseng C.A.Mey*. [Araliaceae; Ginseng Radix Et Rhizoma]   concentration uncertainty   - Root of *Aconitum carmichaelii Debx*. [Ranunculaceae; Aconiti Lateralis Radix Praeparata]   concentration uncertainty   - Root of *Salvia miltiorrhiza Bunge*. [Labiatae; Salviae Miltiorrhizae]   concentration uncertainty   - Mature seeds of *Descurainia sophia (L.) Webb ex Prantl.* [Cruciferae; Descurainiae Semen]   concentration uncertainty   - Tuber of *Alisma plantago-aquatica subsp. orientale (Sam.) Sam.* [Alisaceae; oriental waterplantain rhizome]   concentration uncertainty   - Dry rhizome of *Polygonatum odoratum (Mill.) Druce*. [Liliaceae; Polygonatum]   concentration uncertainty   - Twigs of *Neolitsea cassia (L.) Kosterm.* [Lauraceae; Cinnamomi Ramulus]   concentration uncertainty   - Flower of *Carthamus tinctorius L*. [Compositae; Carthami Flos]   concentration uncertainty   - Root bark of *Periploca sepium Bunge.* [Asclepiaceae; Cortex Periplocae]   concentration uncertainty   - The ripe peel of T*angerine and its cultivars*. [Rutaceae; Citrus × aurantium L.]   concentration uncertainty | Y – Prepared according to the Pharmacopoeia of China, 2020 edition | N |
| Wang HY  2021 | Qili Qiangxin Capsule | Shijiazhuang Yiling Pharmaceutical Co., Ltd. | - Root of *Astragalus mongholicus Bunge.* [Membranaceus Bunge; Astragali Radix.] - concentration uncertainty - Root of *Panax ginseng C.A.Mey*. [Araliaceae; Ginseng Radix Et Rhizoma]   concentration uncertainty   - Root of *Aconitum carmichaelii Debx*. [Ranunculaceae; Aconiti Lateralis Radix Praeparata]   concentration uncertainty   - Root of *Salvia miltiorrhiza Bunge*. [Labiatae; Salviae Miltiorrhizae]   concentration uncertainty   - Mature seeds of *Descurainia sophia (L.) Webb ex Prantl.* [Cruciferae; Descurainiae Semen]   concentration uncertainty   - Tuber of *Alisma plantago-aquatica subsp. orientale (Sam.) Sam.* [Alisaceae; oriental waterplantain rhizome]   concentration uncertainty   - Dry rhizome of *Polygonatum odoratum (Mill.) Druce*. [Liliaceae; Polygonatum]   concentration uncertainty   - Twigs of *Neolitsea cassia (L.) Kosterm.* [Lauraceae; Cinnamomi Ramulus]   concentration uncertainty   - Flower of *Carthamus tinctorius L*. [Compositae; Carthami Flos]   concentration uncertainty   - Root bark of *Periploca sepium Bunge.* [Asclepiaceae; Cortex Periplocae]   concentration uncertainty   - The ripe peel of T*angerine and its cultivars*. [Rutaceae; Citrus × aurantium L.]   concentration uncertainty | Y – Prepared according to the Pharmacopoeia of China, 2020 edition | N |
| Wang L  2019 | Qili Qiangxin Capsule | Shijiazhuang Yiling Pharmaceutical Co., Ltd. | - Root of *Astragalus mongholicus Bunge.* [Membranaceus Bunge; Astragali Radix.] - concentration uncertainty - Root of *Panax ginseng C.A.Mey*. [Araliaceae; Ginseng Radix Et Rhizoma]   concentration uncertainty   - Root of *Aconitum carmichaelii Debx*. [Ranunculaceae; Aconiti Lateralis Radix Praeparata]   concentration uncertainty   - Root of *Salvia miltiorrhiza Bunge*. [Labiatae; Salviae Miltiorrhizae]   concentration uncertainty   - Mature seeds of *Descurainia sophia (L.) Webb ex Prantl.* [Cruciferae; Descurainiae Semen]   concentration uncertainty   - Tuber of *Alisma plantago-aquatica subsp. orientale (Sam.) Sam.* [Alisaceae; oriental waterplantain rhizome]   concentration uncertainty   - Dry rhizome of *Polygonatum odoratum (Mill.) Druce*. [Liliaceae; Polygonatum]   concentration uncertainty   - Twigs of *Neolitsea cassia (L.) Kosterm.* [Lauraceae; Cinnamomi Ramulus]   concentration uncertainty   - Flower of *Carthamus tinctorius L*. [Compositae; Carthami Flos]   concentration uncertainty   - Root bark of *Periploca sepium Bunge.* [Asclepiaceae; Cortex Periplocae]   concentration uncertainty   - The ripe peel of T*angerine and its cultivars*. [Rutaceae; Citrus × aurantium L.]   concentration uncertainty | Y – Prepared according to the Pharmacopoeia of China, 2020 edition | N |
| Wang SK  2020 | Qili Qiangxin Capsule | Shijiazhuang Yiling Pharmaceutical Co., Ltd. | - Root of *Astragalus mongholicus Bunge.* [Membranaceus Bunge; Astragali Radix.] - concentration uncertainty - Root of *Panax ginseng C.A.Mey*. [Araliaceae; Ginseng Radix Et Rhizoma]   concentration uncertainty   - Root of *Aconitum carmichaelii Debx*. [Ranunculaceae; Aconiti Lateralis Radix Praeparata]   concentration uncertainty   - Root of *Salvia miltiorrhiza Bunge*. [Labiatae; Salviae Miltiorrhizae]   concentration uncertainty   - Mature seeds of *Descurainia sophia (L.) Webb ex Prantl.* [Cruciferae; Descurainiae Semen]   concentration uncertainty   - Tuber of *Alisma plantago-aquatica subsp. orientale (Sam.) Sam.* [Alisaceae; oriental waterplantain rhizome]   concentration uncertainty   - Dry rhizome of *Polygonatum odoratum (Mill.) Druce*. [Liliaceae; Polygonatum]   concentration uncertainty   - Twigs of *Neolitsea cassia (L.) Kosterm.* [Lauraceae; Cinnamomi Ramulus]   concentration uncertainty   - Flower of *Carthamus tinctorius L*. [Compositae; Carthami Flos]   concentration uncertainty   - Root bark of *Periploca sepium Bunge.* [Asclepiaceae; Cortex Periplocae]   concentration uncertainty   - The ripe peel of T*angerine and its cultivars*. [Rutaceae; Citrus × aurantium L.]   concentration uncertainty | Y – Prepared according to the Pharmacopoeia of China, 2020 edition | N |
| Xu JF  2020 | Qili Qiangxin Capsule | Shijiazhuang Yiling Pharmaceutical Co., Ltd. | - Root of *Astragalus mongholicus Bunge.* [Membranaceus Bunge; Astragali Radix.] - concentration uncertainty - Root of *Panax ginseng C.A.Mey*. [Araliaceae; Ginseng Radix Et Rhizoma]   concentration uncertainty   - Root of *Aconitum carmichaelii Debx*. [Ranunculaceae; Aconiti Lateralis Radix Praeparata]   concentration uncertainty   - Root of *Salvia miltiorrhiza Bunge*. [Labiatae; Salviae Miltiorrhizae]   concentration uncertainty   - Mature seeds of *Descurainia sophia (L.) Webb ex Prantl.* [Cruciferae; Descurainiae Semen]   concentration uncertainty   - Tuber of *Alisma plantago-aquatica subsp. orientale (Sam.) Sam.* [Alisaceae; oriental waterplantain rhizome]   concentration uncertainty   - Dry rhizome of *Polygonatum odoratum (Mill.) Druce*. [Liliaceae; Polygonatum]   concentration uncertainty   - Twigs of *Neolitsea cassia (L.) Kosterm.* [Lauraceae; Cinnamomi Ramulus]   concentration uncertainty   - Flower of *Carthamus tinctorius L*. [Compositae; Carthami Flos]   concentration uncertainty   - Root bark of *Periploca sepium Bunge.* [Asclepiaceae; Cortex Periplocae]   concentration uncertainty   - The ripe peel of T*angerine and its cultivars*. [Rutaceae; Citrus × aurantium L.]   concentration uncertainty | Y – Prepared according to the Pharmacopoeia of China, 2020 edition | N |
| Yang WB  2021 | Qili Qiangxin Capsule | Shijiazhuang Yiling Pharmaceutical Co., Ltd. | - Root of *Astragalus mongholicus Bunge.* [Membranaceus Bunge; Astragali Radix.] - concentration uncertainty - Root of *Panax ginseng C.A.Mey*. [Araliaceae; Ginseng Radix Et Rhizoma]   concentration uncertainty   - Root of *Aconitum carmichaelii Debx*. [Ranunculaceae; Aconiti Lateralis Radix Praeparata]   concentration uncertainty   - Root of *Salvia miltiorrhiza Bunge*. [Labiatae; Salviae Miltiorrhizae]   concentration uncertainty   - Mature seeds of *Descurainia sophia (L.) Webb ex Prantl.* [Cruciferae; Descurainiae Semen]   concentration uncertainty   - Tuber of *Alisma plantago-aquatica subsp. orientale (Sam.) Sam.* [Alisaceae; oriental waterplantain rhizome]   concentration uncertainty   - Dry rhizome of *Polygonatum odoratum (Mill.) Druce*. [Liliaceae; Polygonatum]   concentration uncertainty   - Twigs of *Neolitsea cassia (L.) Kosterm.* [Lauraceae; Cinnamomi Ramulus]   concentration uncertainty   - Flower of *Carthamus tinctorius L*. [Compositae; Carthami Flos]   concentration uncertainty   - Root bark of *Periploca sepium Bunge.* [Asclepiaceae; Cortex Periplocae]   concentration uncertainty   - The ripe peel of T*angerine and its cultivars*. [Rutaceae; Citrus × aurantium L.]   concentration uncertainty | Y – Prepared according to the Pharmacopoeia of China, 2020 edition | N |
| Wang Q  2021 | Qili Qiangxin Capsule | Shijiazhuang Yiling Pharmaceutical Co., Ltd. | - Root of *Astragalus mongholicus Bunge.* [Membranaceus Bunge; Astragali Radix.] - concentration uncertainty - Root of *Panax ginseng C.A.Mey*. [Araliaceae; Ginseng Radix Et Rhizoma]   concentration uncertainty   - Root of *Aconitum carmichaelii Debx*. [Ranunculaceae; Aconiti Lateralis Radix Praeparata]   concentration uncertainty   - Root of *Salvia miltiorrhiza Bunge*. [Labiatae; Salviae Miltiorrhizae]   concentration uncertainty   - Mature seeds of *Descurainia sophia (L.) Webb ex Prantl.* [Cruciferae; Descurainiae Semen]   concentration uncertainty   - Tuber of *Alisma plantago-aquatica subsp. orientale (Sam.) Sam.* [Alisaceae; oriental waterplantain rhizome]   concentration uncertainty   - Dry rhizome of *Polygonatum odoratum (Mill.) Druce*. [Liliaceae; Polygonatum]   concentration uncertainty   - Twigs of *Neolitsea cassia (L.) Kosterm.* [Lauraceae; Cinnamomi Ramulus]   concentration uncertainty   - Flower of *Carthamus tinctorius L*. [Compositae; Carthami Flos]   concentration uncertainty   - Root bark of *Periploca sepium Bunge.* [Asclepiaceae; Cortex Periplocae]   concentration uncertainty   - The ripe peel of T*angerine and its cultivars*. [Rutaceae; Citrus × aurantium L.]   concentration uncertainty | Y – Prepared according to the Pharmacopoeia of China, 2020 edition | N |
| Yao Y  2021 | Qili Qiangxin Capsule | Shijiazhuang Yiling Pharmaceutical Co., Ltd. | - Root of *Astragalus mongholicus Bunge.* [Membranaceus Bunge; Astragali Radix.] - concentration uncertainty - Root of *Panax ginseng C.A.Mey*. [Araliaceae; Ginseng Radix Et Rhizoma]   concentration uncertainty   - Root of *Aconitum carmichaelii Debx*. [Ranunculaceae; Aconiti Lateralis Radix Praeparata]   concentration uncertainty   - Root of *Salvia miltiorrhiza Bunge*. [Labiatae; Salviae Miltiorrhizae]   concentration uncertainty   - Mature seeds of *Descurainia sophia (L.) Webb ex Prantl.* [Cruciferae; Descurainiae Semen]   concentration uncertainty   - Tuber of *Alisma plantago-aquatica subsp. orientale (Sam.) Sam.* [Alisaceae; oriental waterplantain rhizome]   concentration uncertainty   - Dry rhizome of *Polygonatum odoratum (Mill.) Druce*. [Liliaceae; Polygonatum]   concentration uncertainty   - Twigs of *Neolitsea cassia (L.) Kosterm.* [Lauraceae; Cinnamomi Ramulus]   concentration uncertainty   - Flower of *Carthamus tinctorius L*. [Compositae; Carthami Flos]   concentration uncertainty   - Root bark of *Periploca sepium Bunge.* [Asclepiaceae; Cortex Periplocae]   concentration uncertainty   - The ripe peel of T*angerine and its cultivars*. [Rutaceae; Citrus × aurantium L.]   concentration uncertainty | Y – Prepared according to the Pharmacopoeia of China, 2020 edition | N |
| Zhang YP  2021 | Qili Qiangxin Capsule | Shijiazhuang Yiling Pharmaceutical Co., Ltd. | - Root of *Astragalus mongholicus Bunge.* [Membranaceus Bunge; Astragali Radix.] - concentration uncertainty - Root of *Panax ginseng C.A.Mey*. [Araliaceae; Ginseng Radix Et Rhizoma]   concentration uncertainty   - Root of *Aconitum carmichaelii Debx*. [Ranunculaceae; Aconiti Lateralis Radix Praeparata]   concentration uncertainty   - Root of *Salvia miltiorrhiza Bunge*. [Labiatae; Salviae Miltiorrhizae]   concentration uncertainty   - Mature seeds of *Descurainia sophia (L.) Webb ex Prantl.* [Cruciferae; Descurainiae Semen]   concentration uncertainty   - Tuber of *Alisma plantago-aquatica subsp. orientale (Sam.) Sam.* [Alisaceae; oriental waterplantain rhizome]   concentration uncertainty   - Dry rhizome of *Polygonatum odoratum (Mill.) Druce*. [Liliaceae; Polygonatum]   concentration uncertainty   - Twigs of *Neolitsea cassia (L.) Kosterm.* [Lauraceae; Cinnamomi Ramulus]   concentration uncertainty   - Flower of *Carthamus tinctorius L*. [Compositae; Carthami Flos]   concentration uncertainty   - Root bark of *Periploca sepium Bunge.* [Asclepiaceae; Cortex Periplocae]   concentration uncertainty   - The ripe peel of T*angerine and its cultivars*. [Rutaceae; Citrus × aurantium L.]   concentration uncertainty | Y – Prepared according to the Pharmacopoeia of China, 2020 edition | N |
| Zhang CH  2020 | Qili Qiangxin Capsule | Shijiazhuang Yiling Pharmaceutical Co., Ltd. | - Root of *Astragalus mongholicus Bunge.* [Membranaceus Bunge; Astragali Radix.] - concentration uncertainty - Root of *Panax ginseng C.A.Mey*. [Araliaceae; Ginseng Radix Et Rhizoma]   concentration uncertainty   - Root of *Aconitum carmichaelii Debx*. [Ranunculaceae; Aconiti Lateralis Radix Praeparata]   concentration uncertainty   - Root of *Salvia miltiorrhiza Bunge*. [Labiatae; Salviae Miltiorrhizae]   concentration uncertainty   - Mature seeds of *Descurainia sophia (L.) Webb ex Prantl.* [Cruciferae; Descurainiae Semen]   concentration uncertainty   - Tuber of *Alisma plantago-aquatica subsp. orientale (Sam.) Sam.* [Alisaceae; oriental waterplantain rhizome]   concentration uncertainty   - Dry rhizome of *Polygonatum odoratum (Mill.) Druce*. [Liliaceae; Polygonatum]   concentration uncertainty   - Twigs of *Neolitsea cassia (L.) Kosterm.* [Lauraceae; Cinnamomi Ramulus]   concentration uncertainty   - Flower of *Carthamus tinctorius L*. [Compositae; Carthami Flos]   concentration uncertainty   - Root bark of *Periploca sepium Bunge.* [Asclepiaceae; Cortex Periplocae]   concentration uncertainty   - The ripe peel of T*angerine and its cultivars*. [Rutaceae; Citrus × aurantium L.]   concentration uncertainty | Y – Prepared according to the Pharmacopoeia of China, 2020 edition | N |
| Zhao YQ  2020 | Qili Qiangxin Capsule | Shijiazhuang Yiling Pharmaceutical Co., Ltd. | - Root of *Astragalus mongholicus Bunge.* [Membranaceus Bunge; Astragali Radix.] - concentration uncertainty - Root of *Panax ginseng C.A.Mey*. [Araliaceae; Ginseng Radix Et Rhizoma]   concentration uncertainty   - Root of *Aconitum carmichaelii Debx*. [Ranunculaceae; Aconiti Lateralis Radix Praeparata]   concentration uncertainty   - Root of *Salvia miltiorrhiza Bunge*. [Labiatae; Salviae Miltiorrhizae]   concentration uncertainty   - Mature seeds of *Descurainia sophia (L.) Webb ex Prantl.* [Cruciferae; Descurainiae Semen]   concentration uncertainty   - Tuber of *Alisma plantago-aquatica subsp. orientale (Sam.) Sam.* [Alisaceae; oriental waterplantain rhizome]   concentration uncertainty   - Dry rhizome of *Polygonatum odoratum (Mill.) Druce*. [Liliaceae; Polygonatum]   concentration uncertainty   - Twigs of *Neolitsea cassia (L.) Kosterm.* [Lauraceae; Cinnamomi Ramulus]   concentration uncertainty   - Flower of *Carthamus tinctorius L*. [Compositae; Carthami Flos]   concentration uncertainty   - Root bark of *Periploca sepium Bunge.* [Asclepiaceae; Cortex Periplocae]   concentration uncertainty   - The ripe peel of T*angerine and its cultivars*. [Rutaceae; Citrus × aurantium L.]   concentration uncertainty | Y – Prepared according to the Pharmacopoeia of China, 2020 edition | N |
| Zhao YQ  2020 | Qili Qiangxin Capsule | Shijiazhuang Yiling Pharmaceutical Co., Ltd. | - Root of *Astragalus mongholicus Bunge.* [Membranaceus Bunge; Astragali Radix.] - concentration uncertainty - Root of *Panax ginseng C.A.Mey*. [Araliaceae; Ginseng Radix Et Rhizoma]   concentration uncertainty   - Root of *Aconitum carmichaelii Debx*. [Ranunculaceae; Aconiti Lateralis Radix Praeparata]   concentration uncertainty   - Root of *Salvia miltiorrhiza Bunge*. [Labiatae; Salviae Miltiorrhizae]   concentration uncertainty   - Mature seeds of *Descurainia sophia (L.) Webb ex Prantl.* [Cruciferae; Descurainiae Semen]   concentration uncertainty   - Tuber of *Alisma plantago-aquatica subsp. orientale (Sam.) Sam.* [Alisaceae; oriental waterplantain rhizome]   concentration uncertainty   - Dry rhizome of *Polygonatum odoratum (Mill.) Druce*. [Liliaceae; Polygonatum]   concentration uncertainty   - Twigs of *Neolitsea cassia (L.) Kosterm.* [Lauraceae; Cinnamomi Ramulus]   concentration uncertainty   - Flower of *Carthamus tinctorius L*. [Compositae; Carthami Flos]   concentration uncertainty   - Root bark of *Periploca sepium Bunge.* [Asclepiaceae; Cortex Periplocae]   concentration uncertainty   - The ripe peel of T*angerine and its cultivars*. [Rutaceae; Citrus × aurantium L.]   concentration uncertainty | Y – Prepared according to the Pharmacopoeia of China, 2020 edition | N |
